# The saltmarsh cordgrass *Spartina anglica* has novel genomic features that contribute to abiotic stress tolerance

**DOI:** 10.1093/jxb/erag363

**Published:** 2026-07-21

**Authors:** Rocky D Payet, Shah Md Tamim Kabir, Alejandro Morales-Florez, Althea M E Rose, Lorelei J Bilham, Matthew E Graham, Charlotte Owen, Lucy S Knowles, Xuan Liu, Muhaiminatul Azizah, Georg Pohnert, Anthony J Davy, Jonathan D Todd, J Benjamin Miller

**Affiliations:** School of Biological Sciences, University of East Anglia, Norwich Research Park, Norwich NR4 7TJ, UK; Centre for Microbial Interactions, Norwich Research Park, Norwich NR4 7TJ, UK; School of Biological Sciences, University of East Anglia, Norwich Research Park, Norwich NR4 7TJ, UK; Centre for Microbial Interactions, Norwich Research Park, Norwich NR4 7TJ, UK; School of Biological Sciences, University of East Anglia, Norwich Research Park, Norwich NR4 7TJ, UK; Centre for Microbial Interactions, Norwich Research Park, Norwich NR4 7TJ, UK; School of Biological Sciences, University of East Anglia, Norwich Research Park, Norwich NR4 7TJ, UK; Centre for Microbial Interactions, Norwich Research Park, Norwich NR4 7TJ, UK; School of Biological Sciences, University of East Anglia, Norwich Research Park, Norwich NR4 7TJ, UK; Centre for Microbial Interactions, Norwich Research Park, Norwich NR4 7TJ, UK; School of Biological Sciences, University of East Anglia, Norwich Research Park, Norwich NR4 7TJ, UK; Centre for Microbial Interactions, Norwich Research Park, Norwich NR4 7TJ, UK; John Innes Centre, Norwich Research Park, Norwich NR4 7UH, UK; NERC Environmental Omics Facility, School of Biosciences, University of Sheffield, Sheffield S10 2TN, UK; Centre for Genomic Research, University of Liverpool, Liverpool L69 7ZB, UK; Bioorganic Analytics, Institute of Inorganic and Analytical Chemistry, Friedrich Schiller University Jena, Jena 07743, Germany; Bioorganic Analytics, Institute of Inorganic and Analytical Chemistry, Friedrich Schiller University Jena, Jena 07743, Germany; School of Biological Sciences, University of East Anglia, Norwich Research Park, Norwich NR4 7TJ, UK; School of Biological Sciences, University of East Anglia, Norwich Research Park, Norwich NR4 7TJ, UK; Centre for Microbial Interactions, Norwich Research Park, Norwich NR4 7TJ, UK; MOE Key Laboratory of Evolution and Marine Biodiversity, Frontiers Science Center for Deep Ocean Multispheres and Earth System & College of Marine Life Sciences, Ocean University of China, Qingdao 266003, China; School of Biological Sciences, University of East Anglia, Norwich Research Park, Norwich NR4 7TJ, UK; Centre for Microbial Interactions, Norwich Research Park, Norwich NR4 7TJ, UK; University College Dublin, Ireland

**Keywords:** Abiotic stress tolerance, dimethylsulfoniopropionate, genome, osmolytes, saltmarsh, *Spartina anglica*, transcriptome

## Abstract

The allododecaploid cordgrass *Spartina anglica* thrives in saltmarshes, where it grows vigorously despite facing extreme environmental stresses. The species was formed through a genome duplication of an infertile hybrid in ∼1870, and is today listed amongst 100 of the World’s Worst Invasive Alien Species. Here, we use long-read sequencing to provide a genomic sequence of *S. anglica* and undertake a detailed transcriptomic analysis of different tissues. We demonstrate that *S. anglica* has undergone at least two whole-genome duplication events and exhibits segmental allopolyploidy. We also show that *S. anglica* has evolved novel mechanisms to tolerate abiotic stress, including expansion and neofunctionalization of genes involved in sucrose synthesis, detoxification of reactive oxygen species and heavy metals, and calcium signalling associated with salt, ionic, and nutrient stress. Furthermore, we demonstrate that *S. anglica* produces a diverse array of osmolytes, including dimethylsulfoniopropionate (DMSP), and reveal that a retrotransposon with a unique 55 bp insertion causes high-level expression of the *DMSP-amine oxidase* (*DOX*) gene and contributes to the exceptionally high levels of DMSP production. Taken together, our results highlight the specialised genomic, transcriptomic, and biochemical adaptations that *S. anglica* has evolved to tolerate severe abiotic stresses in saltmarshes.

## Teaser

Genomic and transcriptomic analysis of saltmarsh *Spartina anglica* shows that it produces a diverse range of osmolytes, and identifies a retrotransposon-mediated mechanism responsible for the high-level production of dimethylsulfoniopropionate.

## Introduction

Saltmarshes are significant sites of climate-active gas production and are considered as carbon sinks (Burden *et al.*, 2019). They are highly saline environments that experience routine inundation and waterlogging by sea water (Hou *et al.*, 2024). Saltmarshes are also frequently contaminated by heavy metals and agricultural run-off (Beeftink *et al.*, 1982), and the low-redox state of anaerobic sediments results in the formation of toxic heavy metal sulfides (Rozema *et al.*, 1991). Consequently, saltmarshes are largely occupied by extremophile plant species that can tolerate this range of abiotic stresses (Veldhuis *et al.*, 2019). Among these species, few are as successful as those of the *Spartina* genus (recently integrated into *Sporobolus*, although not widely accepted; Bortolus *et al.*, 2019). Many *Spartina* species are highly invasive (Borges *et al.*, 2021), including *S. anglica* (also called *Sporobolus anglicus*), and can tolerate salt concentrations up to 600 mM NaCl (Vasquez *et al.*, 2006). Indeed, as of 2023, 68 000 ha of land in China alone was occupied by *S. alterniflora* (Qi and Chmura, 2023), an extant parent species of *S. anglica*.

*Spartina anglica* is an allododecaploid member of the *Poaceae* family (Ainouche *et al.*, 2004a). The species is thought to have formed in ∼1870 as the product of a genome duplication of an infertile hybrid (*S. townsendii*), which itself resulted from a cross between the hexaploid species *S. alterniflora* and *S. maritima* (Ainouche *et al.*, 2004a). Considered a xenonative to the UK, *S. anglica* has since been introduced to Asia, Australia, New Zealand, and North America (Bortolus *et al.*, 2019), where it is also considered a highly invasive species and an ‘ecosystem engineer’. As a result, *S. anglica* has been listed as one of the ‘100 of the World’s Worst Invasive Alien Species’ in the IUCN’s Global Invasive Species Database (https://www.iucngisd.org/gisd/100_worst.php), highlighting its global significance.

The halotolerance of *Spartina* species can be attributed to specialist adaptations such as the formation of metal-recreting salt glands (Burke *et al.*, 2000), high-level production of a diverse suite of osmolytes such as glycine betaine (GB) and dimethylsulfoniopropionate (DMSP) (Payet *et al.*, 2024), and the putative expansion of gene families encoding transporters involved in salt stress (Yang *et al.*, 2023). Additionally, *S. anglica* can grow in otherwise anaerobic sediments whose rhizosphere redox potential is elevated by the diffusion of oxygen from aerenchyma in its roots that provide a conduit from the atmosphere (Granse *et al.*, 2022). These specialist adaptations are thought to have contributed to the success of *S. anglica* as an invasive species.

Despite its ecological significance and its remarkable abiotic stress tolerance, no genomic resources and virtually no transcriptomic resources exist for *S. anglica*. Furthermore, to date, few genomes of saltmarsh-inhabiting plant species have been sequenced, and so the effect on a genome of evolving in such an environment remains largely unexplored. Here, using long-read sequencing, we present the first genome sequence for *S. anglica* and evidence for segmental allopolyploidy in this species. We also provide transcriptomic sequencing data for five different tissue types, namely leaf, stem, inflorescence, root, and rhizome. We show that *S. anglica* has evolved novel mechanisms to help it adapt to the severe environmental stresses of saltmarshes, including expansion and neofunctionalization of genes involved in sucrose synthesis, detoxification of reactive oxygen species and heavy metals, and calcium signalling associated with salt, ionic, and nutrient stress. We also demonstrate that *S. anglica* produces a diverse array of osmolytes, including GB, DMSP, proline, proline betaine (stachydrine), ectoine, and dimethylsulfoxonium propionate (DMSOP). Furthermore, we demonstrate that DMSP production occurs at extraordinarily high levels, in part because of a retrotransposon with a unique 55 bp insertion that causes high-level expression of the *DMSP-amine oxidase* (*DOX*) gene in the synthesis pathway. Taken together, our results highlight the various specialised adaptations that *S. anglica* has evolved to tolerate the harsh abiotic stresses of the saltmarsh environment in which it grows.

## Materials and methods

### Plant material and genome sequencing

*Spartina anglica* (syn. *Sporobolus anglicus*) material was collected from a clonal clump at Stiffkey saltmarsh, Norfolk, UK, in 2021, and washed with deionised water to remove surface contamination before being flash-frozen in liquid nitrogen. The material was subsequently ground to a fine powder in liquid nitrogen and 15 g was used for extraction of high-molecular-weight DNA by the NERC Environmental Omics Facility (NEOF), University of Sheffield, UK, using a NuceloBond HMW kit (Macherey-Nagel). Lysis buffer and proteinase K volumes were doubled compared with the manufacturer’s protocol. The sample was incubated for 1 h with gentle agitation every 15 min. The RNase A and binding buffer volumes were also doubled but the rest of the process was completed according to the kit instructions. PacBio libraries were prepared using a SMRTbell prep kit 2.0 (PacBio) and sequenced using a PacBio Sequel IIe platform with a 4-cell SMRT set-up, resulting in a total of 6 257 615 HiFi PacBio reads.

### RNA extraction and sequencing

The plant material was sampled as above and washed with deionised water to remove surface contamination. Plants were then dissected into leaves, stems, inflorescences, roots, and rhizome tissue (*n*=3 plants), after which the individual samples were ground to a fine powder in liquid nitrogen. Total RNA was then extracted using TRI Reagent solution (ThermoFisher Scientific) as per the manufacturer’s instructions followed by cleaning using a Zymo RNA Clean Concentrator™-25 kit. The extracted RNA samples were sent to Novogene where RNA-seq libraries were prepared and sequenced with paired-ends to an average depth of 20 million reads per sample using the Illumina NovaSeq 6000 platform. For quantitative RNA-seq analysis, adapter sequences were first trimmed using Trim Galore (Martin, 2011), after which Salmon (Patro *et al.*, 2017) was used to quantitatively map reads to the coding sequences of our *S. anglica* genome annotation. Differential gene expression was then calculated using DESeq2 (Love *et al.*, 2014), with an adjusted *P*-value of ≤0.05. DESeq2 normalised read numbers were used to visualise expression differences.

### Genome assembly

The genome assembly was generated by NEOF, UK. Briefly, the HiFi PacBio data was assembled using HiFiASM and HiCanu assuming a haplotig number of 6, since *S. anglica* is a hexaploid species that underwent genome duplication (Ainouche *et al.*, 2004b) and has an estimated haploid genome size of 438 Mb (Baumel *et al.*, 2002). This resulted in an assembly of length 2 978 325 964 bp, containing 497 contigs with an N50 of 48 744 091 bp. Contamination was assessed using Blobtools2 (Challis *et al.*, 2020) with default settings; raw read mapping confirmed uniform coverage at a mean depth of 30.43×. Blobtools2 taxonomy assignment returned predominantly no-hit sequences; BLASTn querying of 2000 subsampled reads against the NCBI nucleotide database (https://www.ncbi.nlm.nih.gov/nucleotide/) resolved these as phylogenetically related *Poaceae* (*Oryza sativa*, *Zea mays*, *Panicum* spp., *Sporobolus maritimus*, *Spartina alterniflora*), and were consistent with the GC content (∼45%) of the target organism. No non-plant contaminants were detected at levels warranting removal.

### Gene annotation

The assembled *S. anglica* genome sequences were annotated using MAKER v3.01.03 (Cantarel *et al.*, 2008) as previously described (Card *et al.*, 2019). The initial step involved constructing a *de novo* repeat library using RepeatModeler v2.0.3 (Flynn *et al.*, 2020) and RepeatMasker v4.1.1 (Nansheng, 2004). Subsequently, Trinity v2.11.0 (Grabherr *et al.*, 2011) was employed for the *de novo* assembly of RNA-seq data obtained from the five different tissues, serving as expressed sequence tag evidence. Protein sequences from *Brachypodium distachyon*, *Panicum hallii*, *Arabidopsis thaliana*, and *O. sativa* were retrieved from the UniProt database (https://www.uniprot.org/) for protein homology evidence. Three assemblers were trained using the initial round of maker outputs: Augustus (Stanke *et al.*, 2006), SNAP (Korf, 2004), and GeneMark HMM (Lukashin and Borodovsky, 1998). The gff3 files derived from the first-round MAKER output, along with the trained data, were utilised as inputs for the second round. Custom Perl scripts were employed to extract coding sequences (CDS) and protein sequences from the second-round gff3 file. The genome completeness was evaluated using BUSCO v5.4.7 (Simão *et al.*, 2015) with embryophyte (odb10) datasets. Filtering was then performed to retain only those sequences that had a minimum of one transcript per million (TPM) in the RNA-seq data generated from all replicates of at least one tissue type. The functional annotation of protein coding genes was performed by BLASTp with UniProt/Swiss-Prot proteins (The UniProt Consortium, 2025), NCBI-NR, InterProScan v5.63-95.0 (Jones *et al.*, 2014), and Gene Ontology (GO) as defined by EggNOG (Huerta-Cepas *et al.*, 2019). All of these annotations were merged into a combined gff3 file.

### Orthogroups and gene family duplication

Proteins from the prediction pipeline were grouped into orthogroups using Orthofinder (Emms and Kelly, 2019). The proteomes of *Eragrostis curvula*, *Musa acuminata*, *Zea mays*, *Sporobolus stapfianus* (desiccation-tolerant), and *Sporobolus pyramidalis* (desiccation-sensitive) were used for grouping. Gene-family duplication was then computed using Computation Analysis of gene Family Evolution (CAFE; De Bie *et al.*, 2006) using default parameters, with the phylogenetic species tree and orthogroup count data provided as inputs. Duplication events were then filtered so that only those with an associated *P*-value of ≤0.05 were considered. This list of duplications was then manually examined, and those orthogroups that were implicated in abiotic stress were taken forwards for more detailed analyses.

### Whole-genome duplication analysis

The *S. anglica* protein sequences and CDS nucleotide sequences were provided to both WGDdetector (Yang *et al.*, 2019) and MCScanX (Wang *et al.*, 2012). Both of these tools were run using default parameters and *D*_s_ and *K*_s_ values were retrieved, respectively. These values were then plotted against density and the resulting plots were used to infer genome duplication events.

### Construction of phylogenetic trees

Orthofinder (Emms and Kelly, 2019) was used to establish the phylogeny of *Spartina anglica*. This makes use of the Species Tree Root Inference from Duplication Events (STRIDE) algorithm, which takes all identified orthogroups as an input, constructs gene trees, and then infers the root using duplication events within these.

For phylogenetic trees of specific genes, sequence alignments were performed using MAFFT (Katoh and Standley, 2013) with a maximum of 1000 iterations and the FFT-NS-i model. The resulting alignments were then used as input for IQTree3 (Wong *et al.*, 2025), using 1000 ultrafast bootstraps optimised by nearest-neighbour interchange, and substitution model selection by ModelFinder (Kalyaanamoorthy *et al.*, 2017).

### GO enrichment analyses

Gene ontology enrichment analyses were performed at a tissue level using TopGO (Alexa *et al.*, 2006). The corresponding ‘Gene Universe’ was constructed using the annotated genes.

### Measurement of osmolytes by ultra-HPLC–high-resolution MS

Intact leaves from the three highest and three lowest DMSP-accumulating *S. anglica* plants from Stiffkey saltmarsh were sampled and flash-frozen in liquid nitrogen. Approximately 5 mg (DW) of leaf material was extracted with 500 µl of LC-MS grade methanol and incubated for 30 min. After centrifugation at 16 100 *g* for 15 min, three aliquots of 5 µl of each supernatant were diluted with 95 µl of a mixture of acetonitrile and water (9:1 v/v) containing the internal standards with a final concentration of 5 nM (see below). This solution was then subjected to ultra-HPLC–high-resolution MS (UHPLC-HRMS) analysis.

The analysis of osmolytes via UHPLC-HRMS was based on a previously reported protocol (Azizah and Pohnert, 2022, 2023). Separation was performed on a SeQuant ZIC-HILIC column (2.1×150 mm, 5 µm) equipped with a SeQuant ZIC HILIC guard column (2.1×20 mm, 5 µm) (Merck). The UHPLC mobile phase consisted of high-purity water with 2% LC-MS grade acetonitrile (both Th. Geyer GmbH, Renningen, Germany) and 0.1% LC-MS grade formic acid (Fisher Scientific) (solvent A), and 90% acetonitrile with 10% aqueous LC-MS grade ammonium acetate (1 mM) (LGC Promochem, Wesel, Germany) (solvent B). A gradient elution was performed using an isocratic elution of 100% solvent B for 1 min, followed by a linear gradient from 100% solvent B to 20% solvent B over 5.5 min, then a linear gradient from 20% solvent B to 100% solvent B over 0.6 min, and finally an isocratic re-equilibration at 100% solvent B for 2.9 min. The total run time was 10 min. The column was kept at 25 °C, the flow rate was set at 0.6 ml min^−1^, and the injection volume was 5 µl. Full-scan mode was set from 75–200 *m*/*z* at a resolution of 70 000.

Identification of osmolytes was carried out by comparing the retention times and MS spectra of osmolytes in the samples with the following standards. The commercially available standards used were stachydrine (Phytolab), sarcosine (ABCR GmbH, Karlsruhe, Germany), and choline, ectoine, and proline (all Sigma-Aldrich). DMSP and DMSOP were obtained by synthesis as described in previous studies (Gebser and Pohnert, 2013; Thume *et al.*, 2018).

For quantification, the peak areas of the analytes were compared with calibration curves that were constructed for the internal standards. As internal standards for quantification of DMSP, DMSOP, and ectoine, we used D_6_-DMSP, D_6_-dimethylsulfonioacetate (DMSA), and D_3_-ectoine, respectively. For the rest of the osmolytes, D_3_-gonyol was used. D_6_-DMSP, D_6_-DMSA, D_3_-gonyol, and D_3_-ectoine were acquired by synthesis in our laboratory based on previously published procedures (Gebser and Pohnert, 2013; Thume *et al.*, 2018; Fenizia *et al.*, 2020). For DMSOP, the calibration curve for the area of the molecular ion was *y*=6.75×10^−2^*x*, *r*=0.9944, limit of detection (LOD)=0.6 nM, limit of quantification (LOQ)=2.3 nM; for DMSP, *y*=2.38×10^−1^*x*, *r*=0.9944, LOD=0.6 nM, LOQ=20.1 nM; for ectoine, *y*=2.49×10^−1^*x*, *r*=0.9962, LOD=2.5 nM, LOQ=8.8 nM; for stachydrine, *y*=2.03×10^−1^*x*, *r*=0.9860, LOD=19.1 nM, LOD)=66.1 nM; for choline, *y*=4.61×10^−3^*x*, *r*=0.9968, LOD=55.0 nM, LOQ=193.8 nM; for glycine betaine, *y*=3.85×10^−1^*x*, *r*=0.9890, LOD=2.2 nM, LOQ=7.4 nM; for proline, *y*=4.69×10^−3^*x*, *r*=0.9896, LOD=57.1 nM, LOQ=200.6 nM; and for sarcosine, *y*=2.31×10^−3^*x*, *r*=0.9887, LOD=66.3 nM, LOQ=231.9 nM. All calibration curves were based on *n*=3 replicates and all passed Mandel’s test.

### Testing the function of LTR*^DOX^* in *Nicotiana benthamiana*

Our expression analysis identified a novel ∼2 kb long terminal repeat (LTR) retrotransposon upstream of *DMSP-amine oxidase 6* (*DOX6*), which we named as LTR*^DOX^*. To examine its function, plasmids containing the *35S* minimal promoter driving expression of the firefly luciferase (*LUC*) reporter gene with and without the unique 55 bp sequence found in LTR*^DOX^* (LTR*^DOX^*-p35Sminimal-LUC-t35S and p35Sminimal-LUC-t35S) were constructed, and then assembled into multigene constructs containing a constitutively expressed β-glucuronidase (*GUS*) reporter gene cassette (pAtUBI10-GUS-tNOS) using Golden Gate cloning, as described previously (Tansley *et al.*, 2023). The sequence-verified plasmids were transformed into *Agrobacterium tumefaciens* strain GV3101 and transformation of *Nicotiana benthamiana* leaves was performed by infiltration as previously described (Tansley *et al.*, 2023), with three replicates. At 3 d after infiltration, the luciferase and GUS activities were quantified as previously described (Feike *et al.*, 2019) using a Hidex Sense microplate reader.

## Results

### Sequencing, assembly, and annotation of the *S. anglica* genome

Using the PacBio Sequel IIe platform, a high-quality genome assembly of *S. anglica* with a total of 6.26 million HiFi reads consisting of ∼91.6 Gb (∼30.8×) was generated (Supplementary Table S1). The draft genome assembly length was 2.97 Gb in 497 contigs (N50=48.74 Mb) (Table 1). The expected haploid chromosome number of *S. anglica* is 62 (Renny-Byfield *et al.*, 2010), and consistent with this the largest 62 contigs generated contained 2.89 Gb and represented 96.9% of the total assembled genome (Supplementary Table S2). BUSCO was run on this assembly and reported a 97.7% completeness score and 85.5% duplication.

**Table 1.** *Spartina anglica* genome assembly and annotation statistics

Assembly feature	Value
Assembled genome size (Gb)	2.97
Number of contigs	497
Total length of contigs (bp)	2 978 325 964
N50 of contigs (bp)	48 744 091
N90 of contigs (bp)	28 480 330
GC content (%)	45.18
Transposable elements (%)	71.3
Masked repeat sequence length (bp)	2 140 632 283
Predicted protein-coding genes	91 935
Complete BUSCOs (%)	92.8

N50 and N90 represent the length of the shortest contig for which all contigs of equal or greater length cover for at least 50% and 90% of the assembly, respectively. GC refers to the guanine–cytosine content.

Repeat annotation revealed that 71.3% of the genome is composed of transposable (repetitive) elements (Table 1; Supplementary Table S3). Most of these repetitive elements are made up of retrotransposons (36.4%), which primarily consist of (LTR) retrotransposons of the Ty3/gypsy and Ty1/copia types (23.7% and 10.2%, respectively). The remaining repetitive elements comprise a combination of long interspersed nuclear elements (LINEs; 2.3%), DNA transposons (3.9%), rolling-circle transposons (0.2%), and elements that could not be classified (31.0%). These values for *S. anglica* are consistent with those from the recently published genomes of *S. alterniflora* and *S. maritima* (Salmon *et al.*, 2025).

Annotation of the *S. anglica* draft genome was performed using MAKER (Cantarel *et al.*, 2008), combining *de novo* homology-based evidence and transcriptome data from five different tissue types. MAKER was run twice and filtered based upon evidence of expression, resulting in a total of 91 935 genes that corresponded to 369.6 Mb (12.4%) of the genome. The mean gene length (introns and exons) was 4021 bp, and the number of non-coding tRNAs and rRNAs were 1517 and 6959, respectively (Supplementary Table S4). BUSCO analysis resulted in 92.8% completeness, using embryophyta_odb10 as the reference database (Supplementary Table S5). Of these gene predictions, 82 083 were functionally annotated using EggNOG and the NCBI non-redundant database, and 65 259 and 69 849 were assigned using UniProt and InterProScan, respectively (Supplementary Table S6). All annotated genes were restricted to only 101 contigs of the assembled 497, with Sa017 demonstrating the highest number of genes at 3649 (Fig. 1A; Supplementary Table S7).

**Fig. 1.**
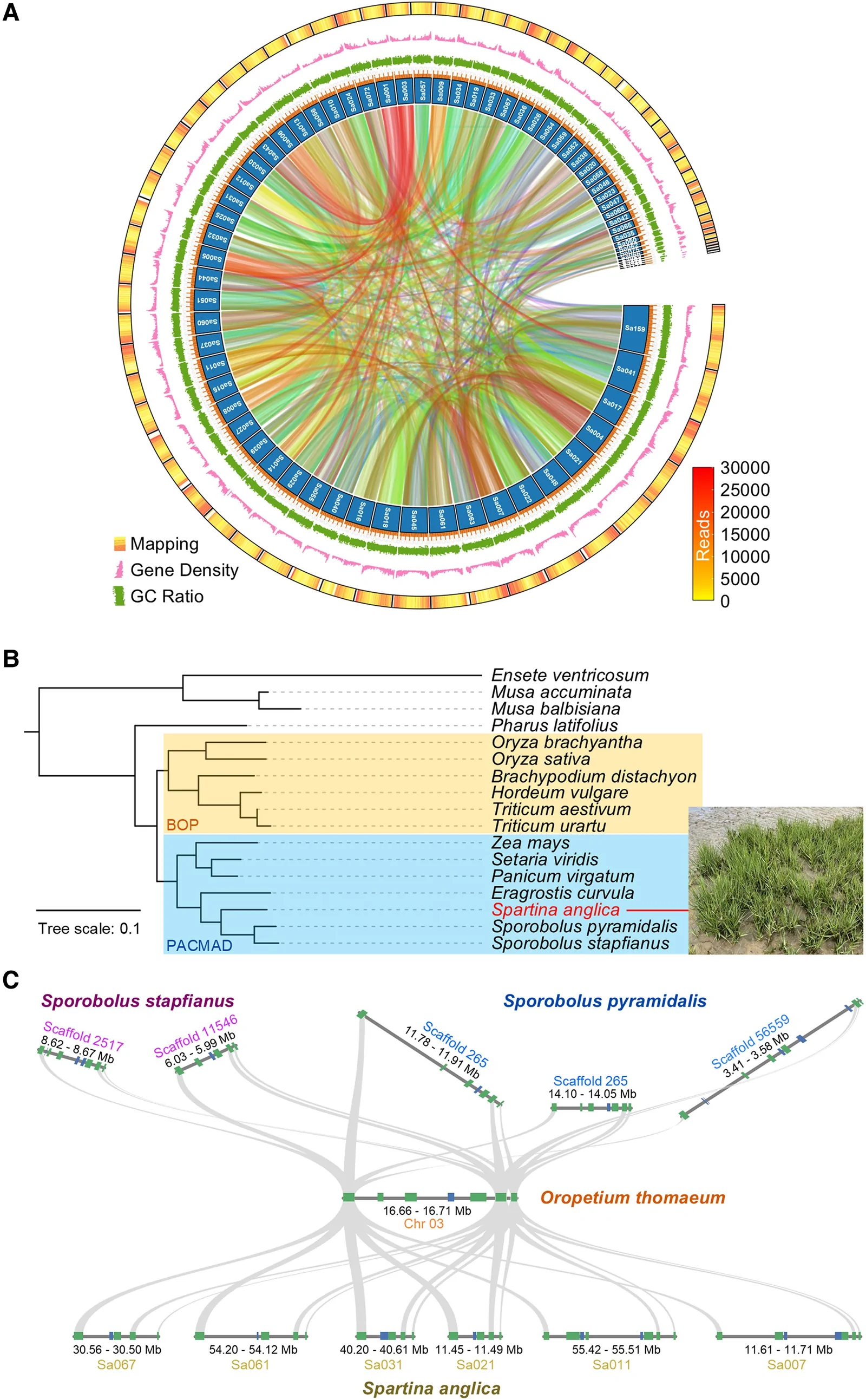
Genomic features, phylogeny, and synteny of *Spartina anglica.* (A) The *S. anglica* genome assembly showing, from outer to inner rings, mapping read counts, gene density (both 100 kb window size), GC content (10 kb window size), contig layout, and syntenic gene relationships. Links among the different contigs are shown. (B) Phylogenetic tree showing the placement of *S. anglica* within the Commelinids clade. The PACMAD and BOP clades of *Poaceae* are indicated. (C) Microsynteny of *S. anglica* with other *Chloridoideae* grass species. The syntenic regions illustrate ploidy levels, with *Oropetium thomaeum* as a diploid, *Sporobolus stapfianus* as a tetraploid, and *Sporobolus pyramidalis* as a hexaploid. *Spartina anglica* is shown as a dodecaploid. Grey lines indicate collinear connections, while gene clusters are represented in green and blue.

Analysis revealed that genes in 68 contigs exhibit collinearity. In total, 69 319 genes were involved in segmental duplication, forming 108 416 collinearity pairs, indicating that 75% of genes evolved from whole-genome duplication. Additionally, 1086 genes have undergone tandem duplication, remaining in close proximity, with 1323 tandem duplication events recorded. A total of 13 178 dispersed genes evolved through transposition events, including replicative, non-replicative, and conservative transpositions. The dataset also included 7582 single-copy (singleton) genes (Supplementary Table S8).

### *Spartina anglica* has undergone at least two whole-genome duplication events and exhibits segmental allopolyploidy

A phylogeny of *S. anglica* was constructed using a combination of genomes representing both the PACMAD and BOP clades of *Poaceae*, and the sister *Musaceae* family (Fig. 1B). The placement of *S. anglica* was consistent with it belonging to the PACMAD clade and to the *Chloridoideae* subfamily. *Spartina anglica* has been subsumed into the genus *Sporobolus*, a decision that has divided scientific opinion (Bortolus *et al.*, 2019). Two established *Sporobolus* genomes have been sequenced more recently (Chávez Montes *et al*., 2022) and were therefore included in our analysis. These two species, *Sporobolus stapfianus* (which is desiccation-tolerant) and *Sporobolus pyramidalis* (desiccation-sensitive), were more closely related to one another than to *S. anglica*, consistent with *Spartina* being a coherent clade within the genus *Sporobolus* in the new taxonomy.

The genome assembly was next examined for evidence of duplication events using two separate tools, MCScanX and WGDdetector, which yielded different but compatible results. Analysis of the *K*_s_ value distribution of the assembly using MCScanX showed a clear peak at ∼1.25 (Supplementary Fig. S1A), consistent with an ancestral whole-genome duplication (WGD) event. There was evidence of this peak in *K*_s_ values generated from WGDdetector, although it was less pronounced (Supplementary Fig. S1B). Interestingly, the data generated with WGDdetector suggested a recent WGD (*K*_s_=0.35), which was not apparent in the MCScanX results. Taken together, these results suggest that *S. anglica* has undergone at least two WGD events, one recent and one ancestral, and that these two events are distinct from the reported WGD event undergone in *S. townsendii* (Ainouche *et al.*, 2004a). Two genomes for the parental species *S. alterniflora* have recently been published (Chen *et al.*, 2024; Hao *et al.*, 2024), and the *K*_s_ values for *S. anglica* that we obtained with MCScanX are consistent with the values derived from the *S. alterniflora* genome, supporting a recent WGD event. However, this was not true of the values we obtained using WGDdetector, which suggested a more ancient WGD event. It is therefore possible that the more ancient WGD seen clearly in Supplementary Fig. S1B arises from the *S. maritima* genome, and indeed a recent study reporting the *S. maritima* and *S. alterniflora* genomes supports this notion (Salmon *et al.*, 2025).

The *Chloridoideae* subfamily of grasses contains the genus *Sporobolus* and is predominantly polyploid. Microsynteny analysis revealed that *S. anglica* exhibited a 6:1 pattern with the diploid grass species *Oropetium thomaeum* (Fig. 1C); in contrast, the two *Sporobolus* species displayed 2:1 and 3:1 patterns. Macrosynteny analysis further highlighted the polyploid nature of *Sporobolus stapfianus* and *Sporobolus pyramidalis*, showing 2:3 patterns that clearly indicated their tetraploid and hexaploid states, respectively (Supplementary Fig. S2), as previously documented (Chávez Montes *et al.*, 2022). Syntenic block analysis demonstrated that 79% of *O. thomaeum* blocks shared a 1:6 pattern with *S. anglica*. Approximately half of *Sporobolus stapfianus* blocks shared a 1:6 pattern, while 34% followed a 2:6 pattern with *S. anglica*. Similarly, *Sporobolus pyramidalis* showed diverse syntenic relationships, with 25% of its blocks following a 3:6 pattern, nearly 50% aligning with a 2:6 pattern, and 17% showing a 1:6 pattern with *S. anglica* blocks. These macrosynteny results suggested a more intricate polyploid structure than the straightforward diploid, tetraploid, hexaploid, or dodecaploid classifications. The findings imply that these species, including *S. anglica*, might exhibit segmental allopolyploidy, with varying degrees of homology among chromosomes derived from different subgenomes.

### Gene expression in *S. anglica* is highly influenced by abiotic stresses associated with the saltmarsh environment

As part of the annotation pipeline, we performed RNA-seq on leaf, stem, inflorescence, root, and rhizome tissues from *S. anglica* plants sampled in the field, and then quantitatively mapped the results to gene annotations to investigate the gene expression profiles. Principal component analysis demonstrated that the transcriptomes of each of the five tissues were distinct (Supplementary Fig. S3), although the root and rhizome tissues clustered together closely. The top 100 most highly abundant transcripts of each tissue were selected and subjected to GO enrichment analysis for biological process terms. The top 20 most highly enriched terms were then examined (Supplementary Table S9). Unsurprisingly, the most abundant transcripts in leaf tissue pertained to photosynthesis. This was also true of inflorescence tissue, despite these two tissues clustering distinctly from one another. There was also a substantial enrichment in inflorescence tissue for the biosynthesis and metabolism of polyamines, such as spermine, which are implicated in developmental processes and environmental stress responses (Blázquez, 2024). Among the top 100 genes in each tissue, many pertained to abiotic stress (Supplementary Table S10), with GO process terms such as response to oxidative stress, response to water deprivation, and response to temperature (Supplementary Table S9). Indeed, when the GO process terms of the top 100 transcripts of each tissue were compared, there were eight enriched process terms common to all five tissues: response to abiotic stimulus, response to inorganic substance, response to light stimulus, response to radiation, response to water deprivation, response to water, response to chemical, and response to toxic substance (Supplementary Fig. S4). Taken together, these results suggested that the transcriptome of *S. anglica* is heavily influenced by abiotic stresses in the environment, particularly those stresses that influence the water status of the plant.

### Expansion of gene families in *S. anglica* probably contributes to tolerance of physiological stress in saltmarshes

Over the course of evolutionary time, gene families are subject to expansions and contractions that, when acted upon by selection, can play a role in adaptation (Demuth and Hahn, 2009). Saltmarshes are high-stress environments, as reflected in the transcriptomic profiles of the various *S. anglica* tissues (Supplementary Fig. S4; Supplementary Tables S9, S10). We therefore hypothesised that the abiotic stresses encountered by *S. anglica* in its native environment might constitute selection pressures sufficient to promote expansion of gene families associated with abiotic stress tolerance. To analyse these events, we ran CAFE using all orthogroups identified between *S. anglica*, *Eragrostis curvula*, *Musa accuminata*, *Z. mays*, *Sporobolus stapfianus*, and *Sporobolus pyramidalis*. This revealed 159 gene families showing expansion or contraction in the *S. anglica* genome (Supplementary Tables S11, S12), with an associated *P*-value of ≤0.05. Of these, 149 families have gained members and 10 have lost them, although the latter category should be treated with caution as the BUSCO score was 93%, indicating some genes might be missing from our assembly. Furthermore, there was a positive correlation between gene expression level and number of gene family members (Supplementary Fig. S5, *R*^2^=0.2878), suggesting that gene family expansion facilitated greater levels of transcription, which could provide a fitness advantage. Amongst the expanded gene families, some of the greatest increases in size were observed in abiotic stress-responsive families (Supplementary Table S11). For example, the sucrose synthase (SUS) family, involved in hypoxia, cold, flooding, and osmotic stress (Wang *et al.*, 2014; Xu *et al.*, 2019; Gurrieri *et al.*, 2020), has doubled in size (10 members in total), and the heat-shock protein 70 family (HSP70), involved in protein folding, temperature stimulus, and response to metals (Usman *et al.*, 2017; Jiang *et al.*, 2020), has also increased (18 members in total). Furthermore, of the top 100 most highly expressed transcripts in each tissue (Supplementary Table S10), *SUS* and *HSP70* family members were common to all five tissue types. In addition to these two families, a number of others involved in stress responses also appeared to have undergone expansion, including salt-sensitivity *NST1-like* genes (Mitsuda *et al.*, 2005), *HSP20* genes (Ji *et al.*, 2019; Guo *et al.*, 2020; Lian *et al.*, 2022), chloride channels (Liu *et al.*, 2023), and universal stress protein family members (Chi *et al.*, 2019) (Supplementary Table S11). Overall, expansion of these abiotic stress-associated gene families probably represents important innovations that together have contributed to the adaptation of *S. anglica* to physiological stress tolerance in saltmarshes and have allowed this species to colonise such an inhospitable environment.

### The SUS family is significantly expanded in *S. anglica* and shows evidence of neofunctionalization

In plants, the SUS protein family falls into three main clades (Stein and Granot, 2019), with members of clade I being stress-responsive and proposed to be the most functionally important (Xu *et al.*, 2019; Gurrieri *et al.*, 2020). One orthogroup of *SUS* family genes has undergone an expansion in *S. anglica* (*P*=0.011), having gained five members relative to closely related species (Supplementary Table S11). To investigate the clade of SUS family proteins in which this expansion occurred, we constructed a phylogenetic tree using characterised SUS protein sequences from Arabidopsis, *O. sativa*, *Solanum lycopersicum*, and *S. anglica* (Fig. 2A). This analysis revealed that the expanded SUS orthogroup in *S. anglica* belonged specifically to the stress-responsive clade I. A subsequent phylogenetic tree was constructed from the clade I SUS family members using sequences from *S. anglica* and closely related species (Fig. 2B; Supplementary Table S13). This revealed that clade I can be further subdivided into two smaller subclades that we call A and B, and which to our knowledge have not been previously described. Interestingly, expansion in the SUS family of *Sporobolus stapfianus* took place in subclade A, whereas the *S. anglica* expansion took place in subclade B, indicating that these family expansion events are unrelated between the two species. Furthermore, within subclade B, a smaller subclade was apparent containing *S. anglica SUS1*/*4*/*5* (subclade B.1), which did not follow the taxonomic relationship between the species represented, suggesting that subclade B.1 is the result of specific expansion in *S. anglica*.

**Fig. 2.**
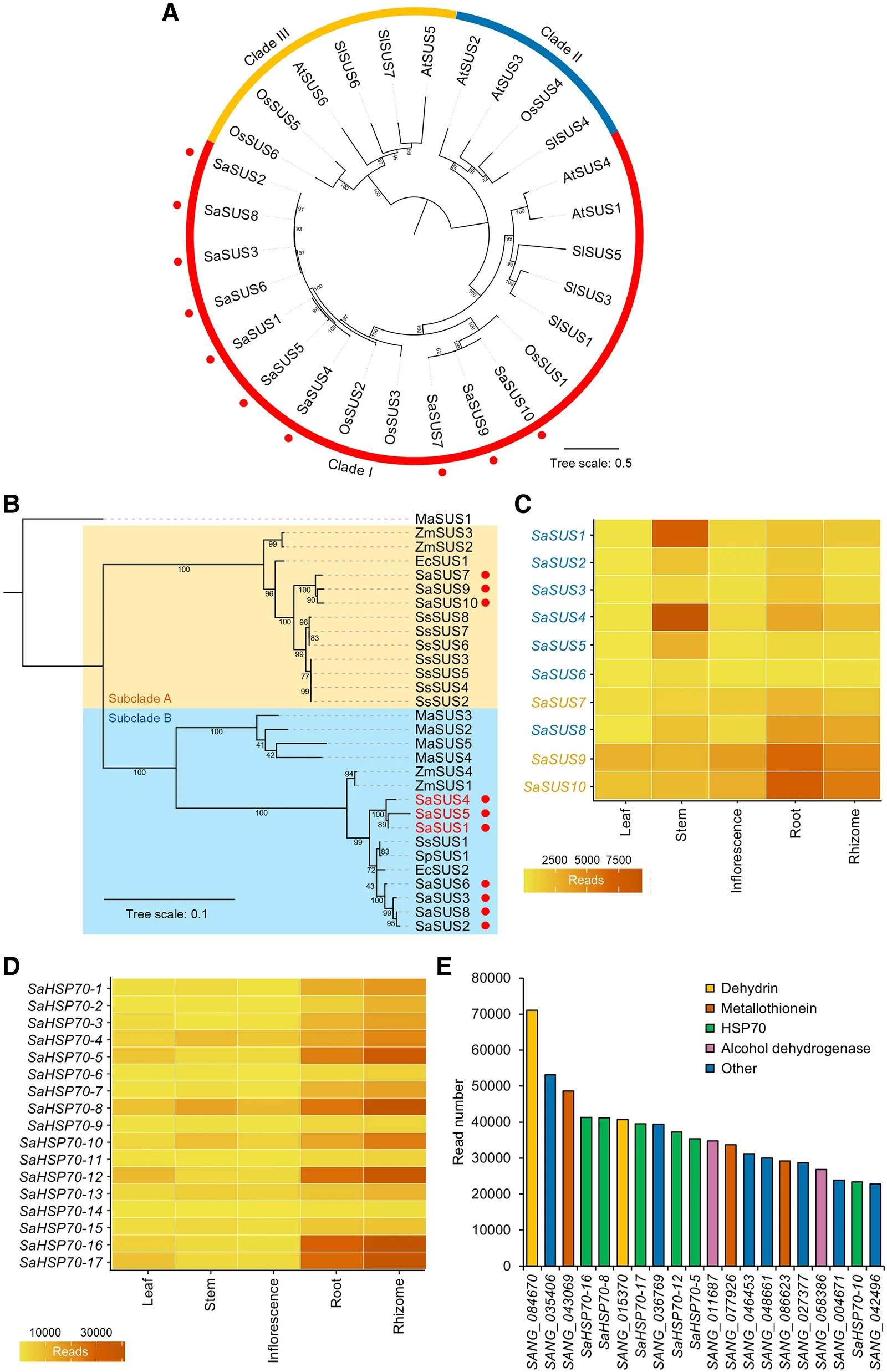
SUS and HSP70 protein families associated with stress tolerance are expanded in *Spartina anglica*. (A) Phylogenetic tree constructed using SUS protein sequences from *Oryza sativa* (Os), *Arabidopsis thaliana* (At), *Solanum lycopersicum* (Sl), and expanded SUS Clade I orthogroup sequences from *S. anglica* (Sa). Colours indicate different clades and dots highlight the *S. anglica* sequences. For visual clarity, Clade II and Clade III orthogroup sequences from *S. anglica* are not shown. (B) Phylogenetic tree constructed using SUS orthogroup sequences from related monocotyledonous plants: *Eragrostis curvula* (Ec), *Musa accuminata* (Ma), *S. anglica* (Sa), *Sporobolus pyramidalis* (Sp), *Sporobolus stapfianus* (Ss), and *Zea mays* (Zm). Dots highlight the *S. anglica* sequences, and the red text indicates the new subclade B.1. Sequences are listed in Supplementary Table S13. (C) Heatmap showing expression of *S. anglica SUS* orthogroup family members in different tissue types. Orange and blue text indicate genes from subclades A and B, respectively. (D) Heatmap showing expression of *S. anglica HSP70* genes in different tissue types. The heatmaps are based on DESeq2 normalised read numbers. (E) Mean read numbers of top 20 most-abundant genes in the transcriptome of *S. anglica* rhizome tissue.

Analysis of expression of the *S. anglica SUS* family members in the different tissues demonstrated that members of subclade A were most highly expressed in roots and rhizomes (Fig. 2C; Supplementary Table S14). The expression of subclade B genes varied between tissues and members of subclade B.1 showed particularly high expression levels in stems. Indeed, *SaSUS4* ranked as the 50th most highly expressed gene in the stem tissue (Supplementary Table S10). It is not clear if members of these subclades display functional differences from one another; however, it is plausible that they are not functionally equivalent given their more distant phylogenetic relationship.

Interestingly, the SUS family appears to have undergone expansion in *Sporobolus stapfianus* as well (Fig. 2B), which also demonstrates considerable abiotic stress tolerance, particularly with regards to desiccation stress (Chávez Montes *et al.*, 2022). However, this expansion was to a lesser extent compared with *S. anglica* (gain of three copies in *Sporobolus stapfianus* versus five in *S. anglica*). The SUS family also demonstrated contraction in *Sporobolus pyramidalis*, which is more sensitive to desiccation than *Sporobolus stapfianus* (Fig. 2B). Taken together, this suggests that the *SUS* gene family is probably central to the physiological stress responses of *S. anglica* growing in saltmarshes.

### The HSP70 family shows an expansion in *S. anglica*

Another orthogroup that has significantly expanded in *S. anglica* encoded HSP70 family proteins (*P*=0.01), having gained six members relative to closely related species (Supplementary Table S11). HSP70 family proteins are ATP-dependent chaperones, implicated in protein folding, and responses to temperature and to metals (Usman *et al.*, 2017; Jiang *et al.*, 2020), and they thus have a well-established role in abiotic stress tolerance in plants. In *S. anglica*, the *HSP70* gene family demonstrated an expansion to 18 members. A phylogenetic tree was constructed with the protein sequences using the same species as for the SUS family tree (Supplementary Fig. S6; Supplementary Table S15). Whilst having more members, the HSP70 family demonstrated lower diversity across the species examined when compared to the SUS family. Additionally, in all cases in *S. anglica*, the *HSP70* family members demonstrated the greatest expression levels in root and rhizome tissues (Fig. 2D; Supplementary Table S16). Indeed, five of the top 10 most highly expressed genes in the rhizome belonged to the expanded *HSP70* family (Fig. 2E), suggesting that these genes might be key to environmental rhizome physiology.

### *Spartina anglica* has many ascorbate peroxidases for detoxifying hydrogen peroxide

Detoxification of reactive oxygen species such as H_2_O_2_ produced in response to abiotic stress is essential for stress tolerance and is achieved by enzymes such as peroxidases. Ascorbate peroxidases (APXs) are type I heme peroxidases that catalyse the H_2_O_2_-dependent oxidation of ascorbate (Pandey *et al.*, 2017; Lazzarotto *et al.*, 2021; Pang *et al.*, 2023). APXs play an essential role in growth regulation and are found across the plant kingdom. Most plant genomes possess multiple copies of the *APX* gene, with eight identified in Arabidopsis, *O. sativa*, and *Z. mays*, and seven in *Solanum lycopersicum* (Narendra *et al.*, 2006; Najami *et al.*, 2008; Pandey *et al.*, 2017; Ribeiro *et al.*, 2017; Qu *et al.*, 2020). Since APXs play significant roles in plants under a variety of abiotic stresses including saline conditions (Diaz-Vivancos *et al.*, 2013), photo-oxidative damage (Fryer *et al.*, 2003; Pignocchi *et al.*, 2003), temperature fluctuations (Zhang *et al.*, 1997; Sato *et al.*, 2011), drought (Rosa *et al.*, 2010), and metal toxicity (Ekmekçi *et al.*, 2008; Rosa *et al.*, 2010; Pandey *et al.*, 2017), we sought to determine whether *S. anglica* has a large number of APXs.

A total of 10 *APX* gene products were identified from the *S. anglica* genome (Fig. 3A; Supplementary Table S17). Plant APXs can be subdivided into several groups depending on subcellular localisation (Pandey *et al.*, 2017; Lazzarotto *et al.*, 2021; Leng *et al.*, 2021; Pang *et al.*, 2023), and eight in *S. anglica* were predicted to be cytosolic. Only SaAPX8 and SaAPX9 were predicted to be peroxisomal. Interestingly, no chloroplastic APXs were identified. The 10 APXs in *S. anglica* were compared with thoses from some of its closest relatives (*Eragrostis curvula*, *Eleusine coracana*, and *Panicum virgatum*, as well as *Z. mays*), and another phylogenetic tree was constructed (Fig. 3B; Supplementary Table S18). This showed that the most significant difference in phylogenetic relationship occurred between the cytosolic and peroxisomal APXs, with no clear phylogenetic relationship between species for the peroxisomal APXs. However, five of the cytosolic SaAPX proteins were most closely related to a single APX from *E. curvula* and *E. coracana* (EcAPX2 and ElcoAPX4, respectively), suggesting expansion of these genes in *S. anglica*. When looking at expression in *S. anglica*, for the majority of *APX* genes there was little difference in the transcript levels across the different plant tissues (Fig. 3C; Supplementary Table S17). However, *APX5* showed higher expression in the roots and rhizome, suggesting that it might play a specific role in decreasing H_2_O_2_ concentrations in these tissues. This is consistent with *APX* genes being more highly expressed in tissues where oxygen is limited, such as below-ground tissues (Blokhina *et al.*, 2001), especially when growing in saltmarsh sediments that are anoxic (Mossman *et al.*, 2020). It was somewhat unexpected that only one out of the 10 *APX* genes showed higher expression in the roots and rhizome of *S. anglica*, but it might be that *SaAPX5* expression is triggered at lower local concentrations of H_2_O_2_ compared with the others, or that there are temporal differences in expression between *SaAPX5* and the others.

**Fig. 3.**
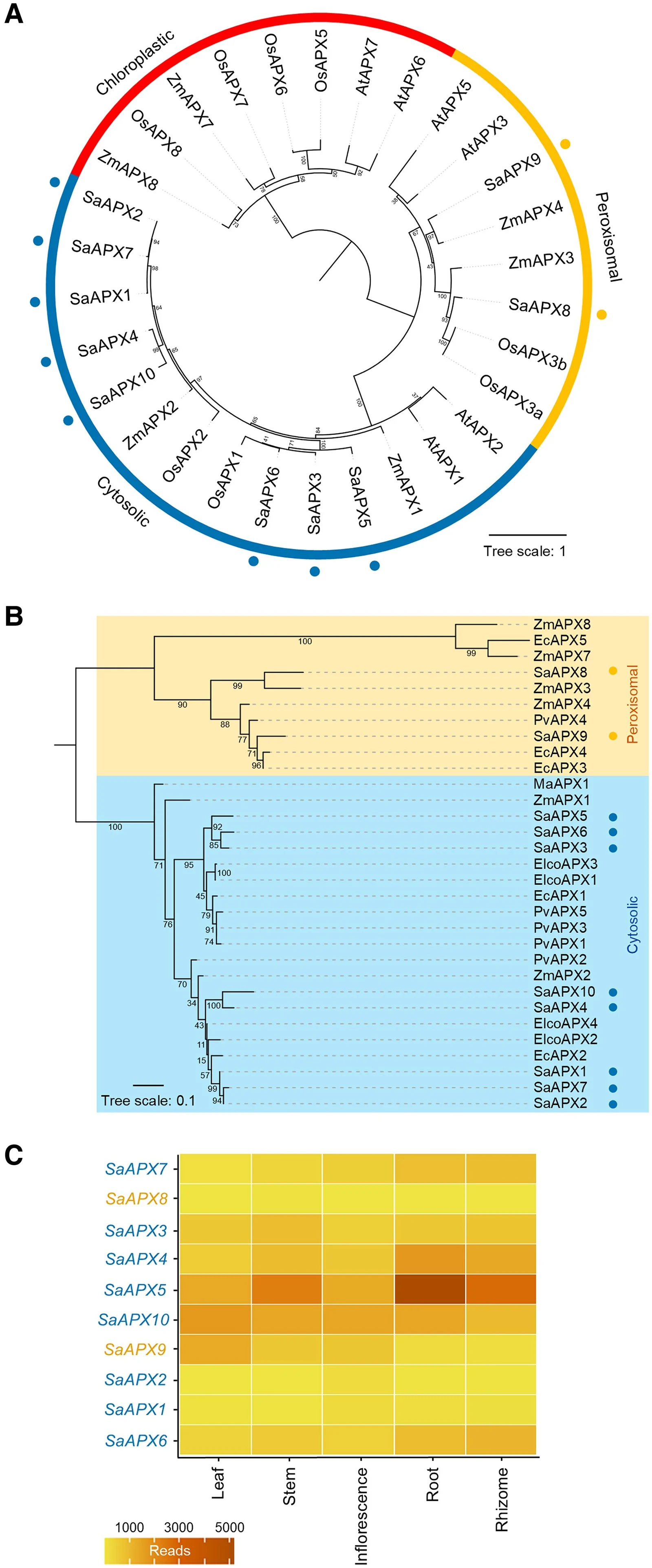
*Spartina anglica* has many stress-responsive ascorbate peroxidase (APX) proteins. (A) Phylogenetic tree constructed using APX protein sequences from *Oryza sativa* (Os), *Arabidopsis thaliana* (At), *Zea mays* (Zm), and *Spartina anglica* (Sa). Colours indicate different clades, which divide according to subcellular protein localisation, and dots highlight *S. anglica* sequences. (B) Phylogenetic tree constructed using APX protein sequences from related monocotyledonous plants: *Eragrostis curvula* (Ec), *Eleusine coracana* (Elco), *Musa accuminata* (Ma), *S. anglica* (Sa), *Panicum virgatum* (Pv), and *Zea mays* (Zm). Dots highlight *S. anglica* sequences. Sequences are listed in Supplementary Table S18. (C) Heatmap showing expression (DESeq2 normalised read numbers) of *S. anglica APX* genes in different tissue types. Orange and blue text indicate genes from the peroxisomal and cytosolic subclades, respectively.

### Proteins involved in calcium-mediated stress signalling are also abundant in *S. anglica* and might allow tolerance to salt, heavy metal ions, and nutrient stress

Calcium signalling mediated by calcium-binding calcineurin B-like proteins (CBLs) and CBL-interacting protein kinases (CIPKs) is essential for responses to abiotic stresses (Batistic and Kudla, 2009), and hence we examined CBLs and CIPKs in *S. anglica*. The protein sequences were identified by BLAST searching the *S. anglica* genome using 10 CBLs and 26 CIPKs from Arabidopsis and 10 CBLs and 33 CIPKs from *O. sativa* as queries. The identified *S. anglica* sequences were then confirmed by determining the presence of key domains: all four EF-hand calcium-binding motifs in CBLs, and the presence of a NAF domain in CIPKs to mediate CBL–CIPK protein interactions (Batistic and Kudla, 2009). In total, 37 CBLs and 69 CIPKs were identified in *S. anglica* (Supplementary Tables S19, S20). A phylogenetic tree showed that there were many CBLs across all groups in *S. anglica* when compared with Arabidopsis and *O. sativa*, but with particular expansion in the Type III transmembrane group (Fig. 4A). AtCBL10 was the only member of this group in Arabidopsis, but in *S. anglica* the group was expanded to include 11 members. AtCBL10 is involved in salt tolerance, probably by interaction with AtCIPK24/SOS2 to activate sodium ion pumping into the vacuole to allow compartmentalisation (Kim *et al.*, 2007). Expansion of this Type III CBL group in *S. anglica* might therefore improve tolerance to the highly saline conditions found in saltmarshes. Analysis of the expression of the *S. anglica CBL* genes showed that *SaCBL4* from this group was highly expressed in all plant tissues (Fig. 4B), suggesting that this gene in particular might play an active role in stress signalling and enabling stress tolerance. *SaCBL12* and *SaCBL21*, part of the Type I lipid-modified, plasma membrane-targeted CBL group (Edel and Kudla, 2015), were highly expressed in the leaf, rhizome, and root tissues. This group contained AtCBL4, also known as SOS3, which is a key protein in the salt tolerance pathway in Arabidopsis (Ali *et al.*, 2023). It is therefore possible that SaCBL12 and SaCBL21 also have important roles in the salt tolerance of *S. anglica*.

**Fig. 4.**
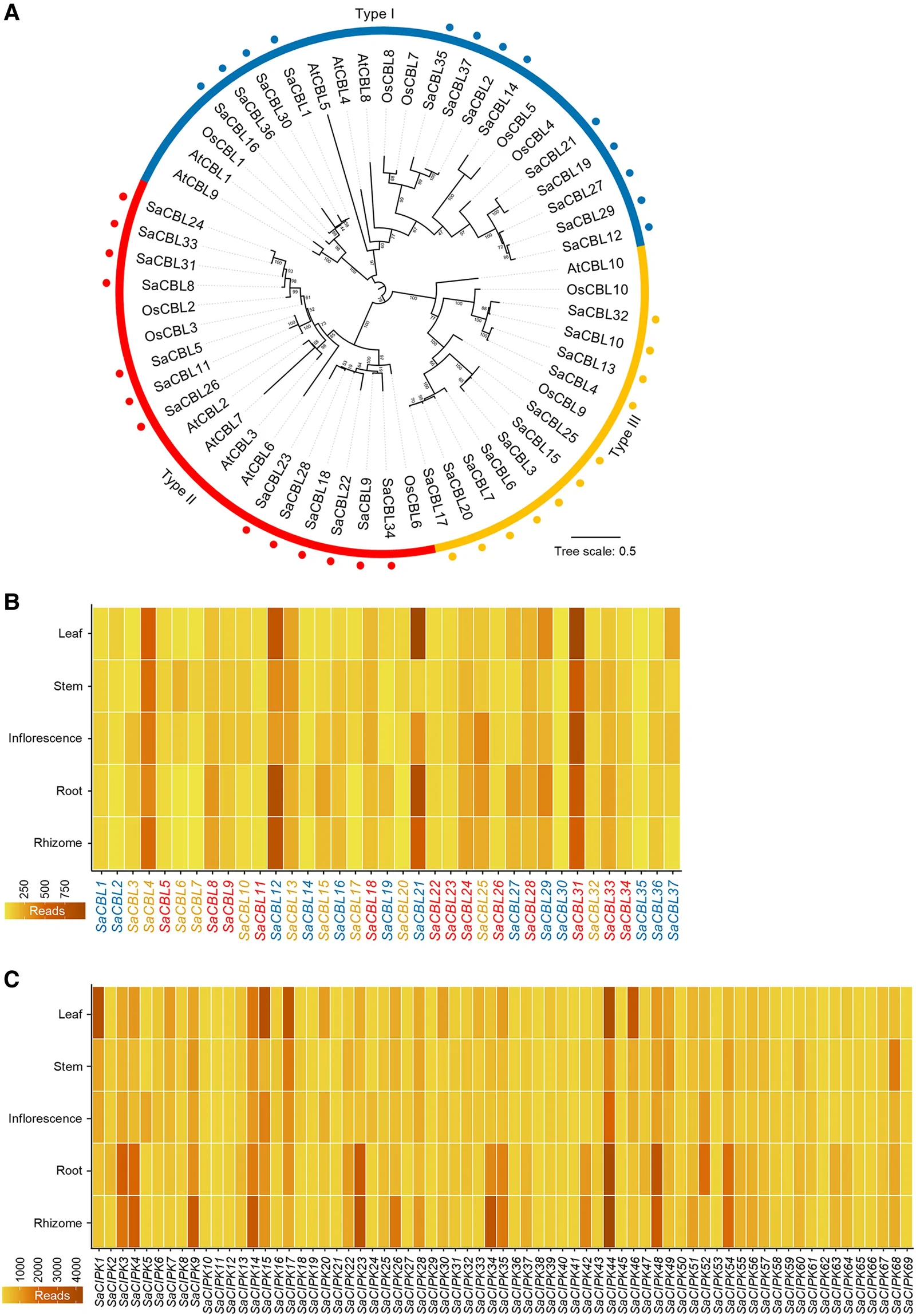
Calcineurin B-like (CBL) and CBL-interacting protein kinase (CIPK) proteins involved in calcium-mediated stress signalling are abundant in *Spartina anglica*. (A) Phylogenetic tree constructed using CBL protein sequences from *Oryza sativa* (Os), *Arabidopsis thaliana* (At) and *S. anglica* (Sa). Colours indicate different clades, which divide according to subcellular protein localisation based on Edel and Kudla (2015): Type I, lipid-modified, plasma membrane-targeted; Type II, lipid-modified, tonoplast-targeted; Type III, transmembrane domain. Dots highlight *S. anglica* sequences. (B) Heatmap showing expression of *S. anglica CBL* genes in different tissue types. Orange, blue, and red text indicate genes from the different clades shown in (A). (C) Heatmap showing expression of *S. anglica CIPK* genes in different tissue types. The heatmaps are based on DESeq2 normalised read numbers.

The many CIPKs that were identified in *S. anglica* were also spread across all groups of the protein family, but particularly large numbers were found within the AtCIPK23 group (Edel and Kudla, 2015), where four Arabidopsis CIPKs and six *O. sativa* CIPKs were expanded to 24 CIPKs in *S. anglica* (Supplementary Fig. S7). AtCIPK23 is a master regulator of ion homeostasis, including heavy metals (Ródenas and Vert, 2021), and is involved in the control of ion channels and transporters for potassium, nitrate, ammonium, manganese, magnesium, cadmium, cobalt, zinc, and iron (Li *et al.*, 2006; Xu *et al.*, 2006; Ho *et al.*, 2009; Maierhofer *et al.*, 2014; Ragel *et al.*, 2015; Tang *et al.*, 2015, 2020; Tian *et al.*, 2016; Straub *et al.*, 2017). Since heavy metals are highly abundant in saltmarsh sediments and soils (Payet *et al.*, 2024), and some nutrients such as nitrogen are found at relatively low concentrations, expansion of the AtCIPK23 group in *S. anglica* might be a direct evolutionary response to these pressures to allow increased stress tolerance to heavy metals and improved regulation of nutrient transport. In support of this, *SaCIPK14*, *SaCIPK34*, *SaCIPK44*, and *SaCIPK54* (all encoding AtCIPK23 group members), showed particularly high expression in the roots and rhizomes (Fig. 4C). As these tissues are important for sensing soil nutrient levels, these four CIPKs might represent important regulators for ion homeostasis signalling pathways in *S. anglica*. Other *S. anglica CIPK* genes that showed high levels of expression in the root included *SaCIPK4* and *SaCIPK23* (from the intronless CIPK group), and *SaCIPK3* and *SaCIPK48* (from the AtCIPK1 group; Edel and Kudla, 2015). SaCIPK4 and SaCIPK23 are related to AtCIPK18, which has a role in lithium ion homeostasis (Zhang *et al.*, 2020), while AtCIPK1 is involved in the Arabidopsis response to deficiencies in nitrate (Su *et al.*, 2024) and phosphate (Lu *et al.*, 2020). This could point towards similar roles for SaCIPK3, SaCIPK4, SaCIPK23, and SaCIPK48 in the regulation of ion homeostasis and nutrient stress.

### *Spartina anglica* produces a range of osmolytes that enhance abiotic stress tolerance in saltmarshes

*Spartina anglica* produces a diverse array of osmolytes at high concentrations, including DMSP, GB, and proline (Cavalieri and Huang, 1981; Payet *et al.*, 2024), some of which are rarely seen at such high levels elsewhere in the plant kingdom (e.g. DMSP; Ausma *et al.*, 2017; Payet *et al.*, 2024). To determine which osmolytes were produced by *S. anglica*, leaf tissue was assayed for a panel of zwitterionic osmolytes known to be produced by other plant species and/or microbes (Supplementary Table S21). Consistent with previous reports, DMSP, GB, and proline accumulated at high concentrations (64, 95, and 7 µmol g^−1^ DW, respectively; Fig. 5A). The relatively low level of proline in relation to the other two compounds (10–14 times lower) was somewhat surprising, suggesting that proline might represent a less important osmolyte in *S. anglica* than in other plant species.

**Fig. 5.**
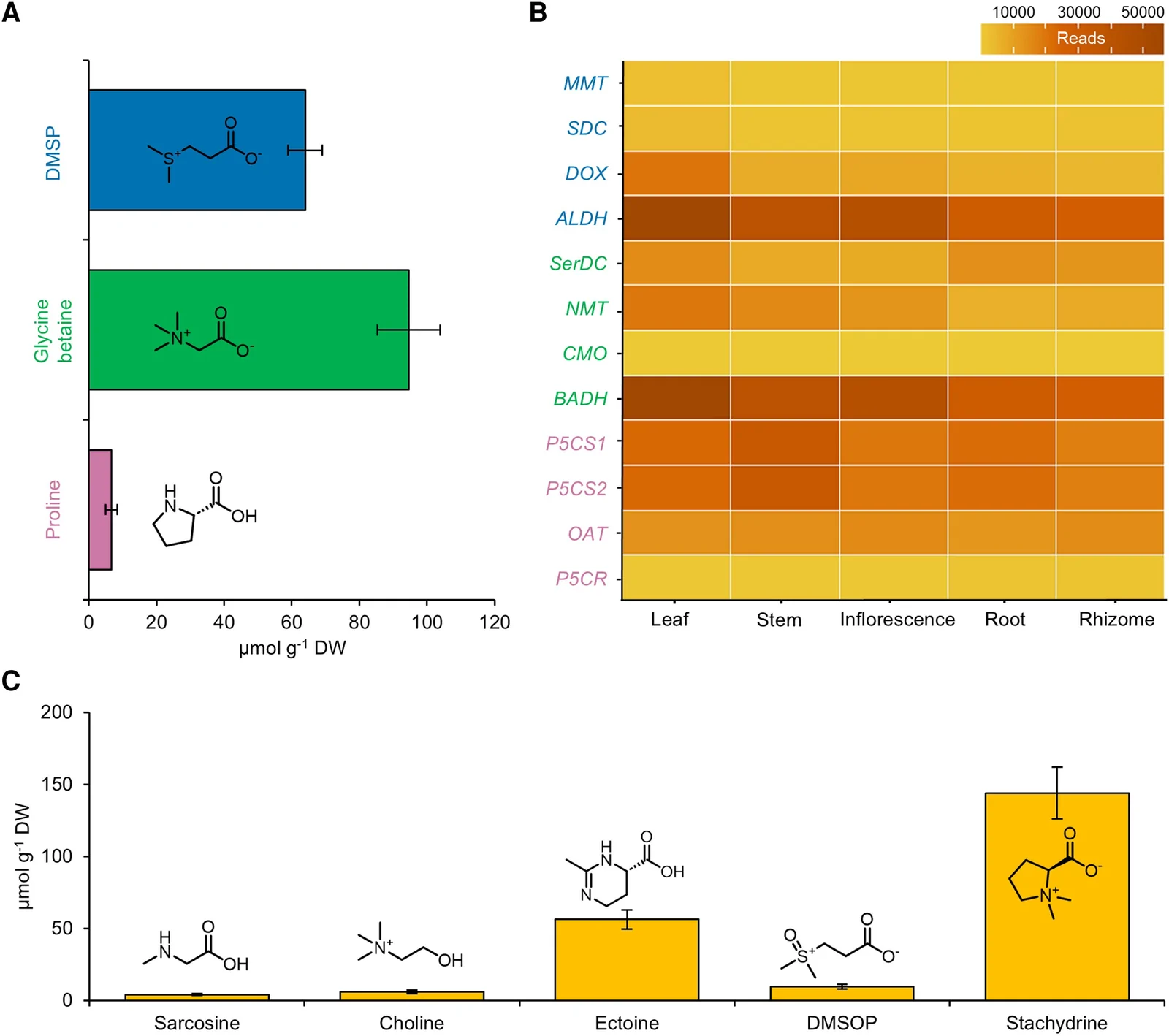
*Spartina anglica* produces a diverse range of osmolytes. (A) Abundance of dimethylsulfoniopropionate (DMSP), glycine betaine, and proline in *S. anglica* leaf tissue. (B) Heatmap showing expression (DESeq2 normalised read numbers) in different tissue types of known biosynthesis genes for the production of DMSP, glycine betaine, and proline, with colours corresponding to (A). (C) Abundance of zwitterionic osmolytes measured in leaf tissue of *S. anglica*. The corresponding chemical structures are shown. Data in (A, C) are means (±SD), *n*=6 independent biological replicates.

We then identified genes encoding the complete known synthesis pathways for DMSP, GB, and proline in the genome assembly (Supplementary Table S22). For the GB synthesis pathway, homologs of the genes encoding enzymes required for each step of the pathway were identified in *S. anglica* by BLAST using *O. sativa* sequences as queries (Luo *et al.*, 2012; Phitaktansakul *et al.*, 2022). In plants, GB is synthesised via choline (Bhuiyan *et al.*, 2007), which itself can act as an osmolyte upon accumulation. However, the choline concentration was 15 times lower than GB in *S. anglica* leaf tissue (Supplementary Table S21), suggesting that choline is mainly an intermediate of GB synthesis rather than an end-product osmolyte in this species. Therefore, the choline synthesis steps were considered to be part of the GB synthesis pathway. Expression analysis showed that genes of the GB and DMSP synthesis pathways were most highly expressed in leaf tissue, whilst genes associated with proline biosynthesis were expressed at similar levels in all five tissue types (Fig. 5B). Despite the concentrations of GB detected in leaves being roughly double those of DMSP (Fig. 5A), there did not appear to be a corresponding increase in the abundance of transcripts of GB synthesis genes. Furthermore, choline monooxygenase (CMO), which bridges the gap between choline and GB biosynthesis, demonstrated very low levels of expression. This suggests either that the GB synthesis enzymes (particularly CMO) display highly favourable kinetics, that substrate availability and/or partitioning plays a greater role in GB accumulation than DMSP, or that other GB synthesis pathways exist in *S. anglica*. Indeed, other pathways have previously been described in halophytic marine bacteria (Liu *et al.*, 2022), which initiate synthesis from glycine rather than choline and proceed through a sarcosine intermediate. Sarcosine was detected in *S. anglica* leaf extracts (4 µmol g^−1^ DW; Supplementary Table S21; Fig. 5C), however no homologs of bacterial glycine-dependent GB synthesis genes (Liu *et al.*, 2022) could be identified by a BLAST search.

The zwitterionic alkaloid proline betaine (stachydrine) was also identified in *S. anglica* (144 µmol g^−1^ DW; Fig. 5C). Previous studies have demonstrated that it accumulates in plants in response to salinity (Hanson *et al.*, 1994; Trinchant *et al.*, 2004) and, coupled with the chemical properties of proline betaine, this supports its role as an osmolyte. Interestingly, proline betaine accumulated at 1.5 times the levels of GB, and its total concentration was approximately equivalent to that of GB and DMSP combined. This suggests that proline betaine might represent one of the main osmolytes in *S. anglica*, which has hitherto been unreported in this genus, or indeed in any grass to the best of our knowledge. To date, no synthesis pathway is known for this compound.

Finally, two further zwitterionic metabolites were also identified in *S. anglica* leaf extracts, namely ectoine and dimethylsulfoxonium propionate (DMSOP) (56 µmol g^−1^ DW and 10 µmol g^−1^ DW, respectively; Fig. 5C). Ectoine has previously been characterised as an osmolyte in halophytic bacteria (Eiberweiser *et al.*, 2015), whilst DMSOP has been identified in marine bacteria and microalgae (Thume *et al.*, 2018) and recently shown to act as an osmolyte that mainly functions in oxidative stress protection (Carrión *et al.*, 2023). To the best of our knowledge, neither of these metabolites have thus far been described in a plant.

### *Spartina anglica* produces high concentrations of DMSP, in part due to a retrotransposon that causes high-level expression of *DMSP-amine oxidase*

*Spartina* species are some of the highest known producers of the osmolyte DMSP (Payet *et al.*, 2024). We therefore sought to characterise its synthesis pathway in greater detail to determine why *S. anglica* produces such high concentrations. The components of the DMSP synthesis pathway *Methionine S-methyltransferase* (*MMT*), *S-methylmethionine decarboxylase* (*SDC*), and *DMSP-amine oxidase* (*DOX*) were most strongly expressed in leaves (Fig. 6A; Supplementary Fig. S8), which correlates with leaves accumulating the highest concentrations of DMSP of all tissues in the plant (Payet *et al.*, 2024). We have previously showed that the SDC enzyme is a key determinant of high-level DMSP production in *S. anglica*, but also that expression of *DOX* is far higher (33–84 times) in *S. anglica* than other plant species (Payet *et al.*, 2024). To investigate why this might be, all *DOX* loci were identified in the *S. anglica* genome assembly. Six distinct loci were identified in total, and expression of *SaDOX6* was found to exceed that of the other five (Fig. 6A; Supplementary Table S23). This analysis was extended to *S. alterniflora*, another *Spartina* species that produces exceptionally high concentrations of DMSP (Chen *et al.*, 2024), where we identified only four *DOX* loci in the genome. As in *S. anglica*, one locus (*SaltDOX3*) showed considerably higher expression than the others (Fig. 6A; Supplementary Table S24). To determine the basis for this novel expression profile, 2 kb windows upstream of each of the six *DOX* genes in *S. anglica* were retrieved and compared. This revealed a ∼2 kb LTR retrotransposon of the gypsy type that was located 651 bp upstream of the transcriptional start site of *SaDOX6* and was completely absent from all the other loci (Fig. 6B). We therefore suspected that this LTR was the cause of the high-level expression and we named it as LTR*^DOX^*. Analysis of the *S. alterniflora* genome also identified LTR*^DOX^* upstream of *SaltDOX3* but not the three other genes.

**Fig. 6.**
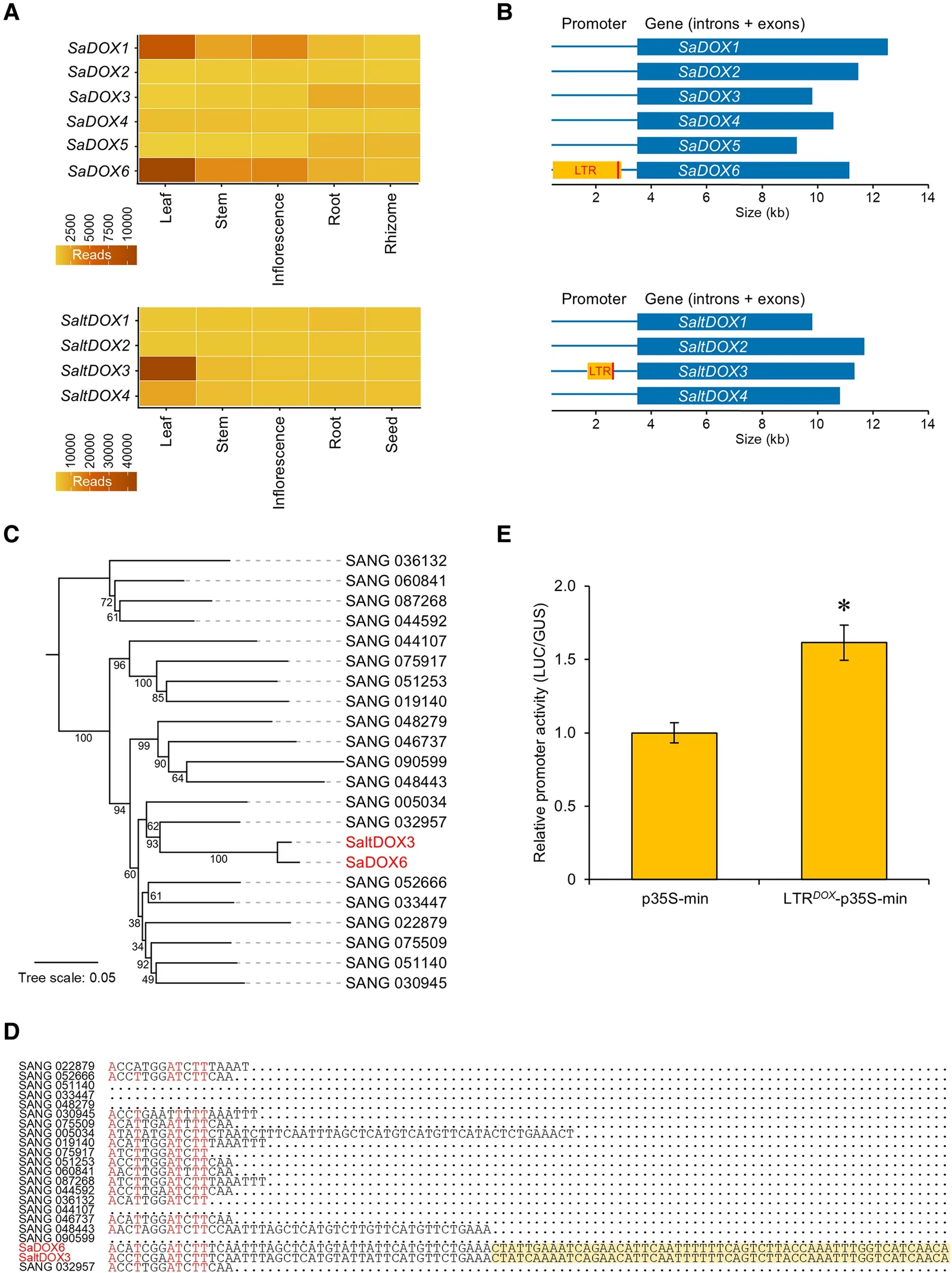
A retrotransposon causes high-level expression of *DOX* and contributes to *Spartina anglica* producing high concentrations of dimethylsulfoniopropionate (DMSP). (A) Heatmaps showing expression (DESeq2 normalised read numbers) of each *DOX* locus in different tissue types of *S. anglica* (Sa) and *S. alterniflora* (Salt). (B) Schematic diagram of each *DOX* locus in the two species. Blue indicates gene sequences (including introns and exons) and orange indicates long terminal repeat (LTR) retrotransposons. Red indicates the 55 bp region found uniquely in LTR*^DOX^* and not in any other genes containing a similar LTR. (C) Phylogenetic tree constructed using LTR sequences from *SaDOX6*, *SaltDOX3*, and the BLAST hits from the *S. anglica* genome where the LTR was detected within 1 kb of a transcriptional start site. (D) Multiple sequence alignment of LTR sequences from (C) showing the unique 55 bp sequence of LTR*^DOX^* (shaded orange). Conserved bases are indicated in red. (E) Quantification of promoter activity in *Nicotiana benthamiana* leaf tissue expressing either the *35S* minimal promoter alone (p35S-min) or the 55 bp sequence of LTR*^DOX^* cloned upstream of the *35S* minimal promoter (LTR*^DOX^*-p35S-min). Data are means (±SD), *n*=3 independent biological replicates.

We next performed a BLAST search using LTR*^DOX^* as a query sequence to determine other potential insertion sites in the *S. anglica* genome that might have a similar high-level expression profile. This resulted in 518 hits, which were then narrowed down to those within a 1 kb window of a transcriptional start site, which yielded only 20 candidates (Fig. 6C). The transcript abundances of these genes were then examined relative to other members of their orthogroup that did not contain this LTR in their promoters. None of the genes showed high-level expression like that of *SaDOX6* (Supplementary Table S25). Comparison of the LTR sequences of the candidate gene promoters with LTR*^DOX^* showed that the sequences were all highly related, but that LTR*^DOX^* occupied its own subclade (Fig. 6C). Sequence alignment revealed that the key difference between LTR*^DOX^* and the other LTRs was the presence of a unique 55 bp sequence towards the 3′-end of LTR*^DOX^*. Once again, this 55 bp sequence was also found in *SaltDOX3* (Fig. 6D). Analysis of the sequence using PlantPAN 4.0 (Chow *et al.*, 2024) identified 49 transcription-factor binding motifs (Supplementary Table S26), which mostly corresponded to MADS, AT-hook, and NF-Y family binding sites. To confirm the impact of this region on gene expression, the LTR*^DOX^* 55 bp sequence was cloned upstream of the *35S* minimal promoter and tested for its ability to drive expression of the *LUC* reporter gene. Including the LTR*^DOX^* 55 bp sequence increased expression by over 50% relative to the control promoter (Fig. 6E). Overall, these results suggested that the LTR*^DOX^* 55 bp sequence is important for high-level expression of *SaDOX*.

## Discussion

Expansion of gene families can result in neofunctionalization, as the introduction of redundancy can free a gene from the consequences of alteration of function within the original process. This can ultimately result in genes that have either new functions or new regulatory patterns. In *S. anglica*, we observed an expansion in the stress-responsive *SUS* family of sucrose synthases. Concurrent with this, we observed a distinct subclade of SUS proteins (clade B.1) that has seemingly emerged in *S. anglica*, as it was absent in closely related species (Fig. 2B). This subclade appeared to have a different expression pattern from the other *SUS* genes, with high expression in the stem. As *S. anglica* occupies saltmarshes and estuaries, the vascular tissues are routinely perfused with highly saline sea water and hence one function that subclade B.1 might serve is to counterbalance the osmotic potential difference in these tissues to minimise water loss. Clade I SUS family members have also expanded in the desiccation-tolerant *Sporobolus stapfianus* and contracted in the desiccation-sensitive *Sporobolus pyramidalis*, indicating that this family might be highly relevant to plant water status.

The HSP70 orthogroup has undergone the largest expansion in *S. anglica*, and this group contained some of the most highly expressed genes in the rhizomes (Fig. 2E). Tolerance to high levels of abiotic stresses such as salinity and inundation are central to the persistence of *S. anglica* rhizomes, and this in turn is critical for the invasive nature of this species; it is therefore possible that expansion of the HSP70 family might represent one of the genetic innovations that underpins this characteristic (Borges *et al.*, 2021). The large abundance of genes involved in the detoxification of reactive oxygen species (Fig. 3) and in calcium signalling (Fig. 4) could also contribute to the ability of *S. anglica* to tolerate high levels of abiotic stress in saltmarshes.

In addition to SUS and HSP70, 147 other orthogroups showed evidence of undergoing expansion (Supplementary Table S11). Given the strong, abiotic selection pressure of saltmarshes, it is reasonable to assume that many of these other expanded families might also convey abiotic stress tolerance, and hence could represent promising candidate genes for future efforts to bioengineer crops that are more tolerant to environmental stress. Indeed, there are already examples emerging of *S. anglica* and *S. alterniflora* genes being used to enhance abiotic stress tolerance in plants (Yang *et al.*, 2023; Payet *et al.*, 2024; Wei *et al.*, 2024). The transcriptome of *S. anglica* displayed many GO process terms related to the water status of the plant (Supplementary Fig. S4) and it is interesting to note that as a C_4_ species, *S. anglica* probably has improved water use efficiency over other saltmarsh C_3_ plants (Monson *et al.*, 2025). Moreover, nitrogen use efficiency is also improved in C_4_ plants (Monson *et al.*, 2025). It is therefore conceivable that the evolutionary links between C_4_ photosynthesis, water use efficiency, and nitrogen use efficiency might have contributed to the ability of *S. anglica* to colonise the waterlogged and nitrogen-poor saltmarsh environment so effectively since its relatively recent formation in ∼1870. The availability of *S. anglica* genomic resources provided by our study might also help identify the molecular mechanisms underpinning other interesting traits found in wetland plant species. For example, comparison between the *S. anglica* and *S. alterniflora* genomes might identify the genetic basis underlying formation of lysigenous aerenchyma, which is reported to be sensitive to flooding in *S. alterniflora* but unaffected in *S. anglica* (Maricle and Lee, 2002). *Spartina anglica* also displayed a rich osmolyte profile, which contained zwitterionic compounds that have not to date been described in plants (e.g. ectoine and DMSOP) and compounds for which biosynthesis enzymes are not currently known in plants (e.g. stachydrine) (Fig. 5C; Supplementary Table S21). Given the high concentrations of these compounds accumulated in *S. anglica*, the genomic data presented here also offer a useful resource for potential discovery of novel pathways. Whether these different osmolytes play different roles or accumulate at different concentrations in various tissue types also remains to be explored in the future.

An outstanding question in the evolution of high-level DMSP synthesis in *Spartina* species has been how the elevated *DOX* expression seen in these species evolved (Payet *et al.*, 2024). Our genome-wide analysis demonstrated that in both *S. anglica* and *S. alterniflora*, most *DOX* transcripts come from one locus, namely *SaDOX6* and *SaltDOX3*, respectively. In both cases, a LTR gypsy family retrotransposon was found to be situated within 1 kb upstream of the transcriptional start site, which we call LTR*^DOX^* (Fig. 6). This LTR contained a 55 bp region that was unique to the *DOX* loci and that could increase gene expression by over 50%. It is possible that a random 55 bp sequence could be sufficient to activate expression of the *35S* minimal promoter, so further work, including mutational approaches, would be interesting to confirm the role of LTR*^DOX^* in increasing gene expression. However, in our previous study, we identified an ethylene-responsive transcription factor (EREBP1) that might regulate DMSP synthesis in *S. anglica* (Payet *et al.*, 2024) and it is interesting to note that an ethylene-responsive AP2/ERF binding motif was identified in the unique 55 bp region of LTR*^DOX^* (Supplementary Table S26). Whether this binding motif is regulated by EREBP1 would be interesting to test in the future. Overall, LTR*^DOX^* represents a good example of how movement of transposable elements can influence gene expression and support the evolution of new traits. Future work to further dissect the *S. anglica* genomic and transcriptomic responses identified here will no doubt yield additional insights into the evolution of this species and the novel adaptations present in the *Spartina* lineage.

## Supplementary Material

erag363_Supplementary_Data

## Data Availability

The genome assembly and annotation files generated in this study have been deposited in the Zenodo repository at https://doi.org/10.5281/zenodo.21354741. The transcriptomic data have been deposited in the NCBI BioProject database (https://www.ncbi.nlm.nih.gov/bioproject) under accession no. PRJNA1495156.

## Supplementary data

The following supplementary data are available at *JXB* online.

Fig. S1. *K*_s_ distributions showing genome duplication events in *Spartina anglica*.

Fig. S2. Macrosynteny among *Oropetium thomaeum* (diploid), *Sporobolus stapfianus* (tetraploid), *Sporobolus pyramidalis* (hexaploid), and *Spartina anglica* (dodecaploid).

Fig. S3. Principal component plot for the RNA-seq data generated from the different tissues of *S. anglica*.

Fig. S4. Venn diagram of the GO terms enriched in the top 100 most highly abundant transcripts in each tissue of *S. anglica*.

Fig. S5. Correlation of gene expression level with the number of gene family members present in *S. anglica*.

Fig. S6. Phylogenetic relationship of Heat Shock Protein 70 (HSP70) from *S. anglica* and related monocotyledonous plants.

Fig. S7. Phylogenetic tree of CBL-interacting protein kinases (CIPKs) in *S. anglica*, *Oryza sativa*, and Arabidopsis.

Fig. S8. Phylogenetic relationships and expression profiles of DMSP synthesis genes from *S. anglica*.

Table S1. Summary of raw PacBio long read statistics used in this study.

Table S2. Length of each contig and cumulative assembly coverage from the PacBio whole-genome sequencing.

Table S3. Number of repetitive/transposable elements present in the *S. anglica* genome.

Table S4. Summary statistics of the predicted protein-coding gene annotations.

Table S5. BUSCO completeness scoring of the *S. anglica* protein annotations.

Table S6. Summary of gene function annotation results.

Table S7. Number of genes annotated on each contig from the PacBio assembly.

Table S8. Numbers of the various types of gene-duplication events detected in *S. anglica*.

Table S9. List of the top 20 most enriched GO process terms represented in the top 100 most highly expressed transcripts in each tissue.

Table S10. Top 100 most highly expressed transcripts in each tissue.

Table S11. Orthogroups that have expanded in *S. anglica* relative to other, closely related grasses.

Table S12. Orthogroups that have contracted in *S. anglica* relative to other, closely related grasses.

Table S13. Protein sequences of the SUS family members used to construct Fig. 2B.

Table S14. Protein sequences and corresponding expression values in the different tissue types for the SUS family members of *S. anglica*.

Table S15. Protein sequences of the HSP70 family members used to construct Fig. S6.

Table S16. Protein sequences and corresponding expression values in the different tissue types for the HSP70 family members of *S. anglica*.

Table S17. Protein sequences and corresponding expression values in the different tissue types for the APX family members of *S. anglica*.

Table S18. Protein sequences of the APX family members used to construct Fig. 3B.

Table S19. Protein sequences and corresponding expression values in the different tissue types for the CBL family members of *S. anglica*.

Table S20. Protein sequences and corresponding expression values in the different tissue types for the CIPK family members of *S. anglica*.

Table S21. UHPLC-HRMS measurements of the various zwitterionic osmolytes present in *S. anglica*.

Table S22. DMSP, GB, and proline biosynthesis pathway genes identified in *S. anglica* with aggregated total abundance of all copies in each tissue type.

Table S23. Protein sequences and corresponding expression values for the DMSP synthesis pathway components in the different tissue types of *S. anglica*.

Table S24. Protein sequences and corresponding expression values for the DOXs in the different tissue types of *S. alterniflora*.

Table S25. IDs and corresponding expression values in the different tissue types of *S. anglica* for the genes containing LTR*^DOX^* within a 1 kb window of the transcriptional start site.

Table S26. Transcription factor binding motifs in the unique 55 bp LTR*^DOX^* sequence.
