# Supplementary material for: The saltmarsh cordgrass Spartina anglica has novel genomic features that contribute to abiotic stress tolerance
Source: J Exp Bot. 2026 Jul 21;77(18):6256–75. doi: 10.1093/jxb/erag363 (PMC13621321; doi:10.1093/jxb/erag363)
Supplement: Supplementary file 1 [file erag363_supplementary_data.zip › Supplementary_Data_updated_FINAL.pdf]

SUPPLEMENTARY DATA

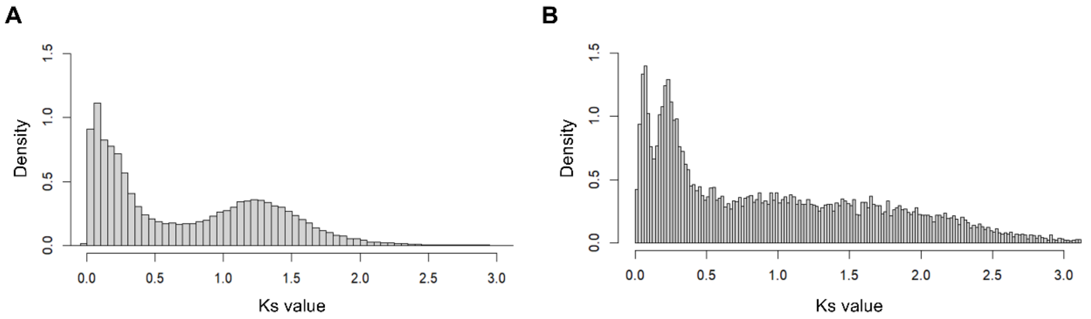

Supplementary Figure S1: Ks distributions showing genome duplication events in *Spartina anglica*
A. Ks distribution of *S. anglica* orthogroups produced using MCScanX, showing evidence of an ancestral whole genome duplication event at Ks = 1.25. **B.** Ks distribution of *S. anglica* orthogroups produced using WGDdetector, showing evidence of a recent whole genome duplication event at Ks = 0.35.

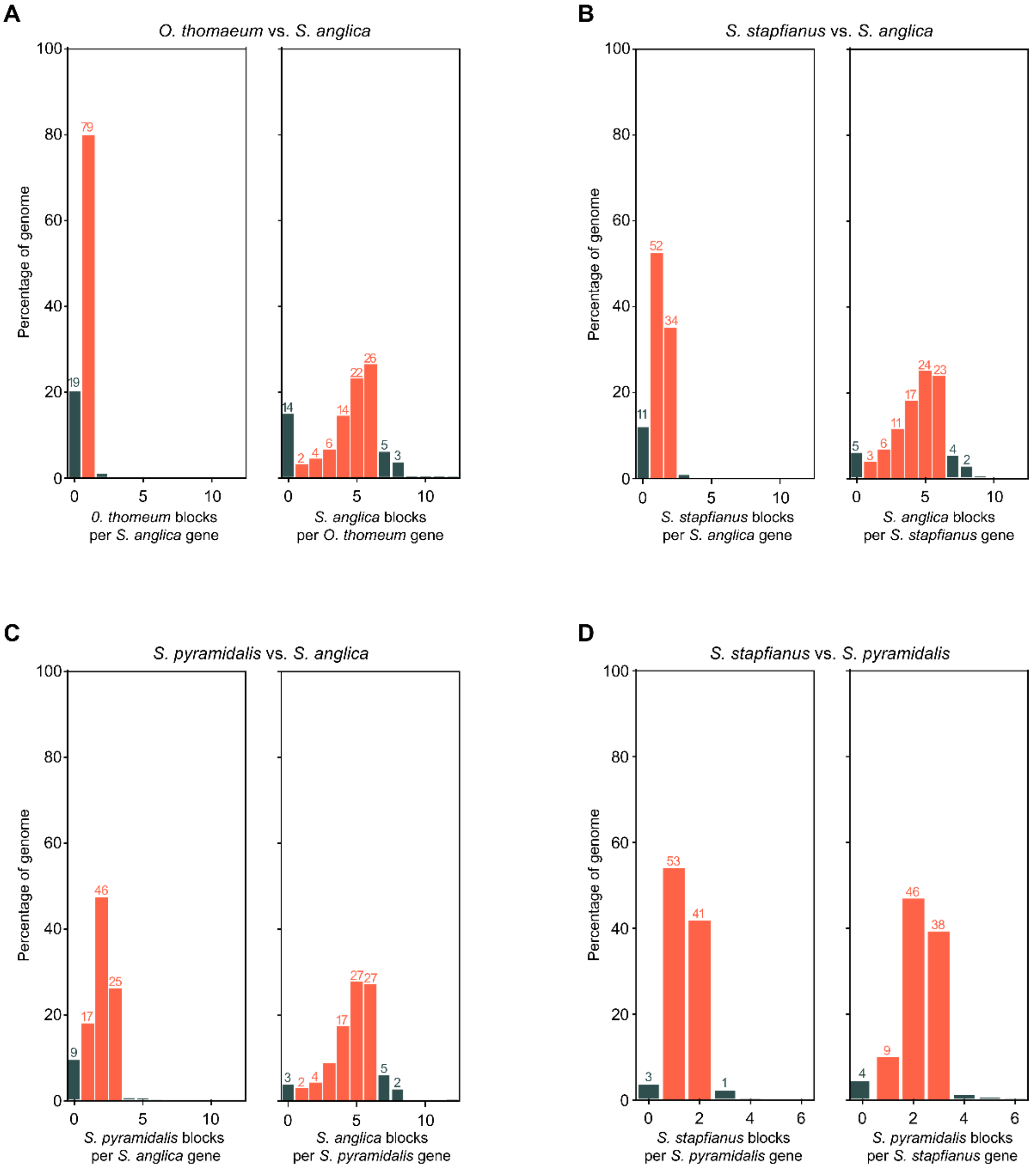

Supplementary Figure S2: Macrosynteny among *Oropetium thomaeum* (diploid), *Sporobolus stapfianus* (tetraploid), *Sporobolus pyramidalis* (hexaploid) and *Spartina anglica* (dodecaploid)

Syntenic block analysis as follows: **A.** *O. thomaeum* vs. *S. anglica* (1:6); **B.** *S. stapfianus* vs. *S. anglica* (2:6); **C.** *S. pyramidalis* vs. *S. anglica* (3:6); and **D.** *S. stapfianus* vs. *S. pyramidalis* (2:3). These relationships illustrate a clear 1:2:3:6 pattern among *O. thomaeum*, *S. stapfianus*, *S. pyramidalis* and *S. anglica*, respectively.

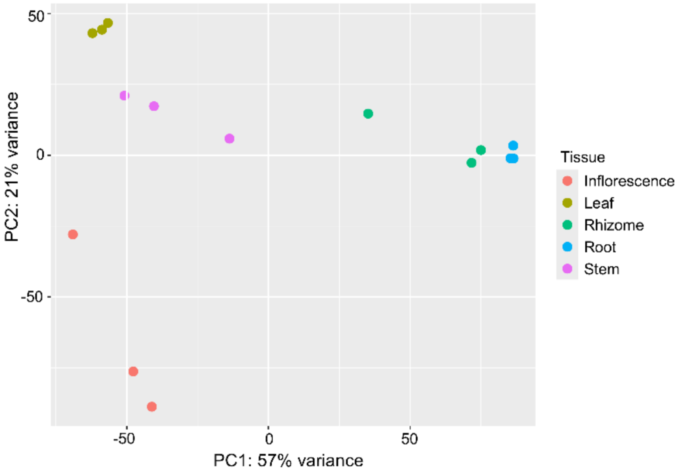

Supplementary Figure S3: Principal component plot for the RNA-seq data generated from the different tissues of *S. anglica*

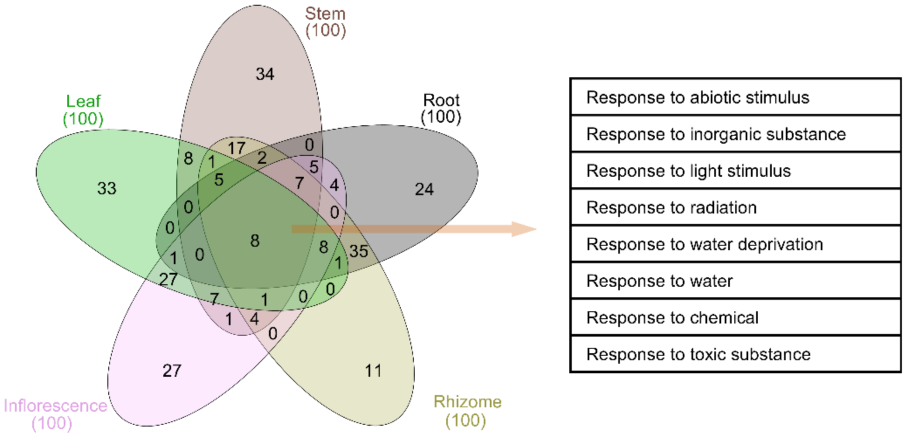

Supplementary Figure S4: Venn diagram illustrating the GO terms enriched in the top 100 most highly abundant transcripts in each tissue of *S. anglica*
Process terms are shown for the eight overlapping GO terms that are common to all five tissue types.

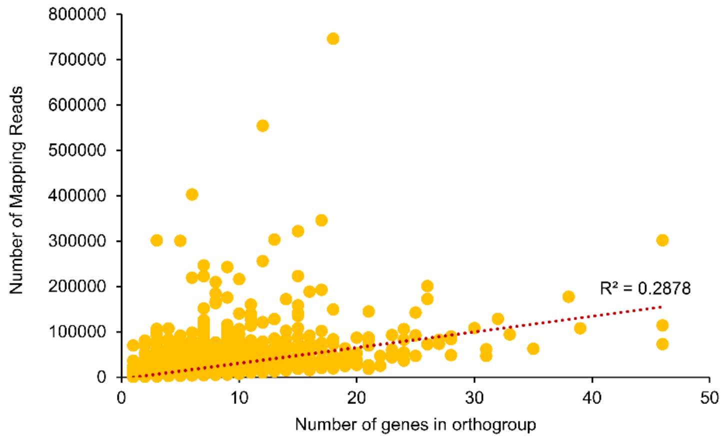

Supplementary Figure S5: Correlation of gene expression level with the number of gene family members present in *S. anglica*

Graph shows the total transcript abundance (sum of expression of all orthogroup members) plotted against the number of genes present in each orthogroup.

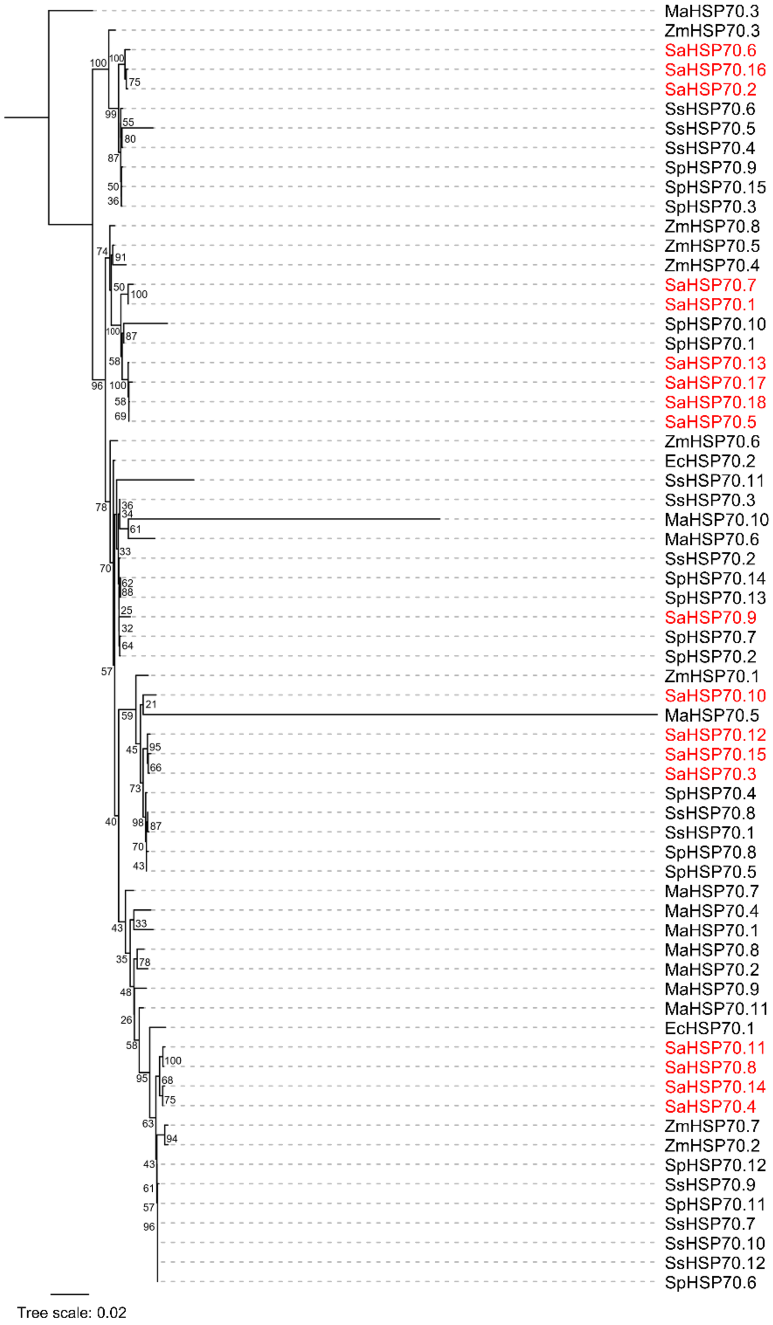

Supplementary Figure S6: Phylogenetic relationship of Heat Shock Protein 70 (HSP70) from *S. anglica* and related monocotyledonous plants

Phylogenetic trees constructed using Heat Shock Protein 70 sequences from *Spartina anglica* and related monocotyledonous plants (*Eragrostis curvula* (Ec), *Musa accuminata* (Ma), *Spartina anglica* (Sa), *Sporobolus pyramidalis* (Sp), *Sporobolus stapfianus* (Ss), and *Zea mays* (Zm)). Red text indicates *S. anglica* sequences.

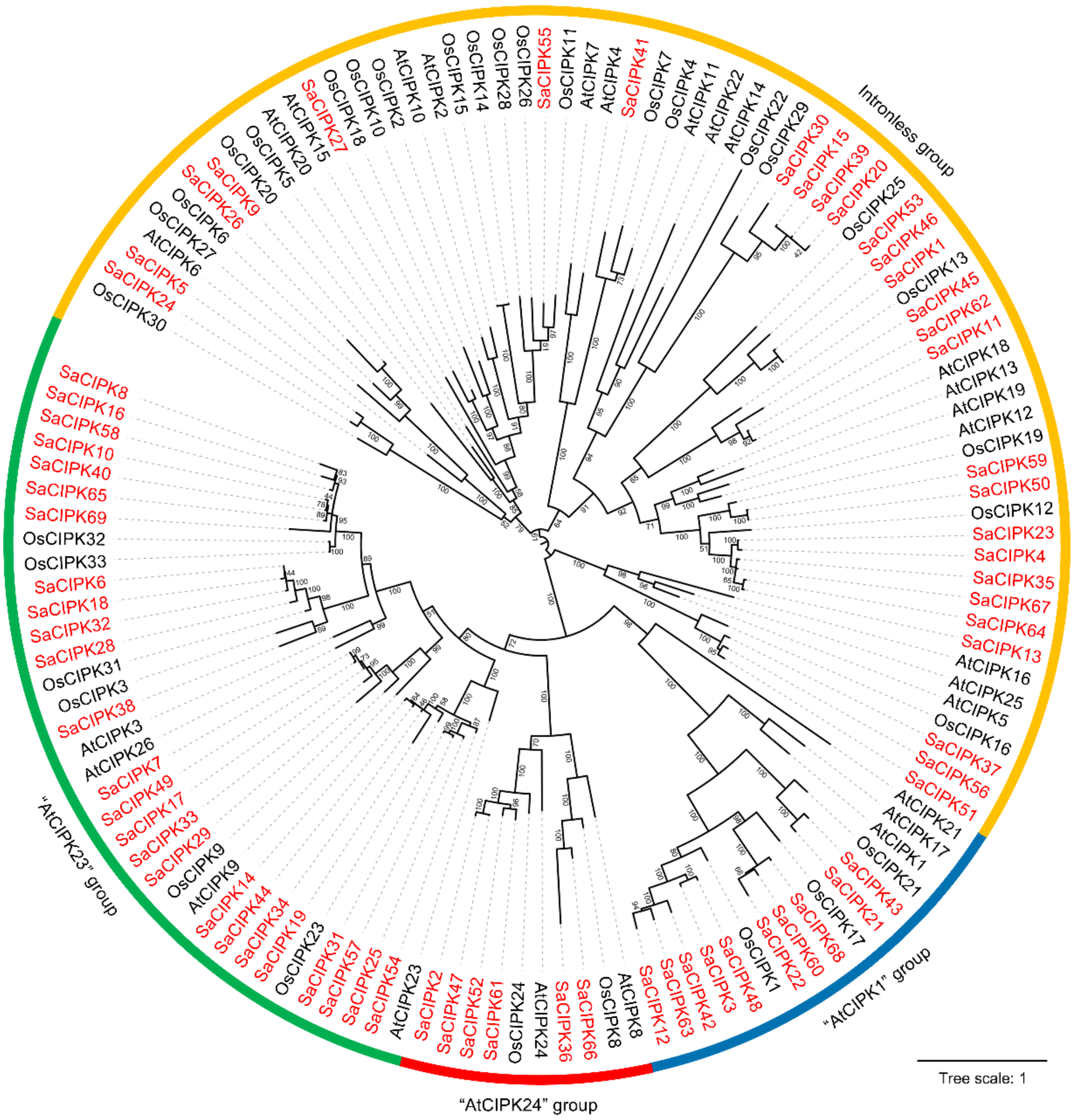

Supplementary Figure S7: Phylogenetic tree of CBL-interacting protein kinases (CIPKs) in *S. anglica*, *Oryza sativa*, and Arabidopsis

Phylogenetic tree constructed using *CIPK* protein sequences from *Oryza sativa* (Os), *Arabidopsis thaliana* (At) and *Spartina anglica* (Sa). Colours indicate the clades, which divide according to groupings based on Edel and Kudla (2015): Intronless CIPK group, “AtCIPK1” group, “AtCIPK23” group, and “AtCIPK24” group. Red text indicates sequences from *S. anglica*.

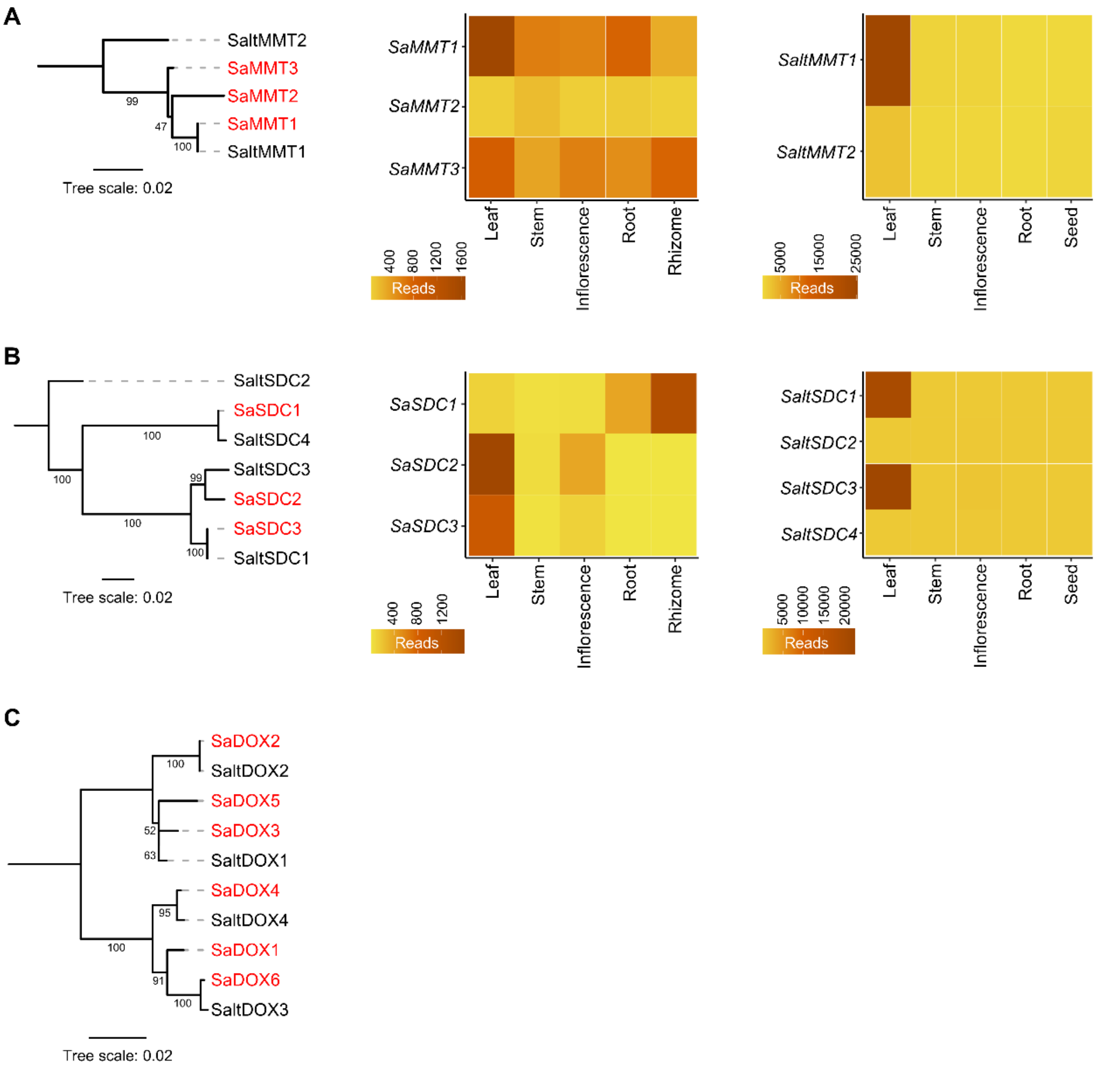

Supplementary Figure S8: **Phylogenetic relationships and expression profiles of DMSP synthesis genes from *S. anglica***

Phylogenetic trees constructed using DMP synthesis protein sequences, and corresponding heatmaps showing expression of each gene locus in different tissue types of *Spartina anglica* (Sa) and *Spartina alterniflora* (Salt). Red text indicates sequences from *S. anglica*. Panels represent: **A.** MMT; **B.** SDC; and **C.** DOX. *DOX* expression data is shown in Figure 6A.
